# A Voting-Enhanced Dynamic-Window-Length Classifier for SSVEP-Based BCIs

**DOI:** 10.1109/TNSRE.2021.3106876

**Published:** 2021-09-06

**Authors:** Hadi Habibzadeh, James J. S. Norton, Theresa M. Vaughan, Tolga Soyata, Daphney-Stavroula Zois

**Affiliations:** Department of Electrical and Computer Engineering, University at Albany, State University of New York, Albany, NY 12222 USA; National Center for Adaptive Neurotechnologies, Office of Research and Development, United States Department of Veterans Affairs, Stratton VA Medical Center, Albany, NY 12208 USA; Department of Electrical and Computer Engineering, University at Albany, State University of New York, Albany, NY 12222 USA; National Center for Adaptive Neurotechnologies, Office of Research and Development, United States Department of Veterans Affairs, Stratton VA Medical Center, Albany, NY 12208 USA; National Center for Adaptive Neurotechnologies, Office of Research and Development, United States Department of Veterans Affairs, Stratton VA Medical Center, Albany, NY 12208 USA; Department of Electrical and Computer Engineering, University at Albany, State University of New York, Albany, NY 12222 USA; Department of Electrical and Computer Engineering, George Mason University, Fairfax, VA 22030 USA; Department of Electrical and Computer Engineering, University at Albany, State University of New York, Albany, NY 12222 USA; National Center for Adaptive Neurotechnologies, Office of Research and Development, United States Department of Veterans Affairs, Stratton VA Medical Center, Albany, NY 12208 USA

**Keywords:** Brain-computer interface, steady-state visual evoked potentials, minimum energy combination, filter bank canonical correlation analysis, maximum synchronization index

## Abstract

We present a dynamic window-length classifier for steady-state visual evoked potential (SSVEP)-based brain-computer interfaces (BCIs) that does not require the user to choose a feature extraction method or channel set. Instead, the classifier uses multiple feature extraction methods and channel selections to infer the SSVEP and relies on majority voting to pick the most likely target. The classifier extends the window length dynamically if no target obtains the majority of votes. Compared with existing solutions, our classifier: (i) does not assume that any single feature extraction method will consistently outperform the others; (ii) adapts the channel selection to individual users or tasks; (iii) uses dynamic window lengths; (iv) is unsupervised (i.e., does not need training). Collectively, these characteristics make the classifier easy-to-use, especially for caregivers and others with limited technical expertise. We evaluated the performance of our classifier on a publicly available benchmark dataset from 35 healthy participants. We compared the information transfer rate (ITR) of this new classifier to those of the minimum energy combination (MEC), maximum synchronization index (MSI), and filter bank canonical correlation analysis (FBCCA). The new classifier increases average ITR to 123.5 bits-per-minute (bpm), 47.5, 51.2, and 19.5 bpm greater than the MEC, MSI, and FBCCA classifiers, respectively.

## I. INTRODUCTION

BRAIN-COMPUTER interfaces (BCIs) are devices that enable people to control computer systems using brain activity [1]. Because they require little to no voluntary motor control, BCIs can help people with severe motor deficits (e.g., locked-in syndrome) to communicate [2]. They may also have applications for healthy people [3]–[6].

Steady-state visual evoked potential (SSVEP)-based BCIs for text-entry (i.e., *SSVEP-based spellers*) are one common type of BCIs [7]. In these systems, users are presented with a set of stimuli, each flashing at a unique frequency. Attention to one of these stimuli elicits changes in brain activity at the fundamental and higher harmonic frequencies of the flashing—an SSVEP—that can be measured using electroencephalography (EEG). These changes in EEG can be quantified and allow a *classifier* to infer the stimulus the user is attending to (i.e., the *target* that the user wants to select) [8]. Each stimulus is mapped to one or more characters; sequential selection of targets allows users to input text [7].

The design of the classifier is critical to the performance of SSVEP-based spellers. Ideally, the classifier correctly infers the target (i.e., has perfect *accuracy*) immediately (i.e., with zero *delay*) after the user starts attending to it. In actual practice, SSVEPs are small and embedded in EEG signals that are contaminated with noise from multiple sources (e.g., movement, muscle activity, etc.) [9], [10]; classifiers often misidentify targets and input incorrect text. Users have to correct these mistakes, decreasing text-entry rates. Improving the performance of an SSVEP-based speller requires designing a classifier that identifies targets as accurately and as quickly as possible. This entails many design choices, including:

- *Feature-Extraction Method:* There are many ways to identify SSVEPs embedded in noisy EEG signals; each works in a slightly different way. The minimum energy combination (MEC) method minimizes the signal-to-noise ratio (SNR) of nuisance signals [11]; the maximum synchronization index (MSI) maximizes the synchronization index between a template of an SSVEP and set of EEG signals [12]; and canonical correlation analysis (CCA) finds the maximum possible correlation between templates of an SSVEP and a set of EEG signals [13]. Although filter bank CCA (FBCCA) (a CCA variant [14]) generally performs better than other methods, no single method uniformly outperforms the others (See Fig. S5).
- *Channel Selection:* SSVEP-based BCIs generally include EEG signals recorded from as many as 128 electrodes placed at different locations on the scalp. The goal of channel selection is to find the set of EEG signals that Maximizes the performance of the classifier. Adding more channels does not always improve performance [15] (Fig. S1); thus, the best set of channels is often determined through offline analysis [14], which itself has limitations, including the inability to produce a global solution due to inter-subject differences (especially in those with injuries or illness), and the failure to account for changes in the scalp distributions of SSVEPs that can occur during a task [16].
- *Window Length:* The window length defines the number of samples to collect before making a classification. When choosing a window length, there is a trade-off between classification accuracy and classification delay. Longer window lengths improve classification accuracy, but also increase classification delay. There are two approaches to balancing this trade-off. Fixed window-length classifiers collect the same number of samples before making a classification. They are simpler to implement and typically determine the best window length using offline analysis [17], [18]. On the other hand, dynamic window-length classifiers adjust the window length over time [19]–[21]. For example, the classifier introduced by da Cruz *et al.* [20] increased or decreased the window length by analyzing the number of times the participant used the “delete” character. Classifiers that use dynamic window lengths are more complicated to implement but may provide a better trade-off between classification accuracy and classification delay.

In this paper, we introduce a new classifier that does not require the user to choose a feature extraction method, channel selection, or window length. Instead, it uses voting to determine the target based on multiple feature extraction methods and many different channel sets. Individual votes are obtained by using every permutation of the feature extraction method and channel selection to infer the target. The classifier then identifies the target as the stimulus with the majority of the votes. If, however, none of the stimuli receives the majority of the votes, the classifier dynamically extends the window length until this requirement is met.

Our classifier has multiple advantages over existing SSVEP-classifiers: (i) it does not assume that any single feature extraction method will uniformly outperform all the others; (ii) it adapts its channel selection depending on the individual user and the task; (iii) its window length is dynamic; and (iv) it is unsupervised (i.e., does not require any offline training). Collectively, these characteristics make our classifier particularly advantageous for clinical applications, where there is neither the time nor the technical expertise to precisely tune the classifier.

The rest of this paper is organized as follows. Section II describes our classifier. Section III describes the experiments we completed to compare our classifier with three existing classifiers, and Section IV provides the results of these experiments. We then discuss the results (Section V) and present our conclusions (Section VI).

## II. CLASSIFIER

To describe our classifier, we first explain how we perform feature extraction and channel selection in Section II–A and Section II–B. We then describe how we dynamically adjust the window length in Section II–C. Finally, we provide the algorithm for our classifier in Section II–D.

### A. Feature Extraction

Let *E* be the set of $N_{e}\in \mathbb{N}$ EEG signals. A feature extraction method Φ(*E*) uses the EEG signals in *E* (typically, by linearly combining them) to extract features. For a given set *E* and its power set P(*E*) (assume P(*E*) excludes the empty set), let $E_{i}^{\Phi }\subseteq \text{P}(E)$ be the set of all subsets of *E* that lead to the selection of target *i* (i.e., a vote for target *i*) using the feature extraction method Φ. For target *i*, we define *ψ*_*i*_(Φ, *E*) as:

(1) $$\psi_{i}(\Phi ,E)=\frac{\left|E_{i}^{\Phi }\right|}{\left|\text{P}(E)\right|}=\frac{\left|E_{i}^{\Phi }\right|}{2^{N_{e}}-1},$$

where |·| is the set cardinality operator. We observe that 0 ≤ *ψ*_*i*_(Φ, *E*) ≤ 1 for all *i*’s and $\sum_{i}\psi_{i}(\Phi ,E)=1$. As a numerical example, if EEG signals are collected using eight electrodes (indexed from one to eight), then *N*_*e*_ = 8. In this case, there are at most 255 (i.e., 2^8^ – 1) possible channel selections. Assume that only channel selections {1, 2, 3} and {7, 8} result in a vote for target *i* using feature extraction method Φ. Thus, $E_{i}^{\Phi }=\{\{1,\;2,\;3\},\;\{7,\;8\}\}$. Consequently, $\left|E_{i}^{\Phi }\right|=2$ and *ψ*_*i*_(Φ, *E*) = 2*/*255.

Equation (1) can be applied to virtually all feature extraction methods (e.g., MEC, MSI, CCA, and FBCCA). Additionally, it can be generalized into multi-dimensional spaces, enabling users to avoid the design decision for the selection of a feature extraction method.

Let = [Φ_1_,Φ_2_, … ,Φ_*K*_ ] be a vector of *K* different feature extraction methods (or the same feature extraction method but with different parameters). We can use Eq. (1) to compute *ψ*_*i*_ (Φ, *E*) = [*ψ*_*i*_(Φ_1_, *E*), … ,*ψ*_*i*_(Φ_*K*_, *E*)]. For these *K*-feature extraction methods, we define the extracted feature of target *i* as:

(2) $$\psi_{i}(\Phi ,E)=\frac{1}{K}\times \|\Psi (\Phi ,E){\|}_{\ell }^{2},$$

where ‖ · ‖_*ℓ*_ is the *ℓ*-norm operator. Herein, we use the Euclidean norm.

### B. Dynamic Channel-Selection

In the standard 10–10 EEG electrode placement system, 21 electrodes cover the occipital and parietal regions of the scalp. For a set *E* that includes all of these electrodes, |P(E)| = 2^21^–1 (excluding the empty set). Hence, computing *ψ*_*i*_(Φ, *E*) per Eq. (1) becomes computationally prohibitive. Instead, we estimate *ψ*_*i*_(Φ, *E*) as follows:

(3) $${\hat{\Psi }}_{i}(\Phi ,E)=\frac{\left|{\hat{E}}_{i}^{\Phi }\right|}{\left|{\hat{\text{P}}}_{R}(E)\right|},$$

where ${\hat{\text{P}}}_{R}(E)$ is computed by randomly selecting *R* elements of *P*(*E*) with equal probability. ${\hat{E}}_{i}^{\Phi }$ is the set of all channel selections in ${\hat{\text{P}}}_{R}(E)$ that result in a vote for target *i* using the feature extraction method Φ. Computing ${\hat{\Psi }}_{i}(\Phi ,E)$ per Eq. (3) only requires *R* (vs. $2^{N_{e}}-1$) votes.

If target *i* is the correct target (i.e., the target the user is attending to), then ${\hat{\Psi }}_{i}(\Phi ,E)$ measures the probability of selecting the (often non-unique) channel selection that leads to a vote for the correct target using feature extraction method Φ. For example, if no channel selection results in a vote for target *i* (i.e., ${\hat{\Psi }}_{i}(\Phi ,E)=0$), the probability of selecting the correct channel selection is zero. Likewise, if all possible channel selections result in a vote for target *i*, then the probability of selecting the correct channel selection is one. More likely scenarios fall between these two extreme cases. Because the correct target is unknown during classification, we assume the target with the largest ${\hat{\Psi }}_{i}(\Phi ,E)$ is the correct target.

### C. Dynamic Window Length

The feature ${\hat{\Psi }}_{i}(\Phi ,E)\in [0,\;1]$ represents the ratio of votes for target *i*. Our classifier dynamically increases the window-length until one of the targets obtains the majority of votes. The classifier uses pre-defined threshold values (denoted by *τ* ∈ [0, 1]) to determine whether a target has obtained the majority of votes (e.g., *τ* = 0.5 instructs the classifier to select the target that collects 50% of votes. If no target has enough votes, the classifier extends the window length).

### D. Algorithm

The algorithm for our classifier has six steps:

1. The classifier receives the number of targets *N*, *K* different feature extraction methods, a vector of threshold values *τ* (one threshold for each window length), and the number of additional samples *W* that it collects when extending the window length. The classifier also chooses *R* different random channel selections.
2. It then classifies the signal using each channel selection and each feature extraction method. A counter vector (*V*) of size *K* × *N* keeps track of the number of votes that each target receives.
3. After iterating through all *K* × *R* cases, the classifier normalizes *V* by dividing its elements by *R*.
4. The classifier uses Eq. (2) to obtain *ψ*_*i*_(Φ, *E*) for each *i* ∈ {1, 2, … , *N*}.
5. If *ψ*_*i*_(Φ, *E*) ≤ *τ* for all *i*, the classifier collects *W* more samples and goes to step 2. Else;
6. The classifier returns target i* as the output of the classifier such that:
  (4) $$i*=\underset{i}{\mathrm{argmax}\;}{\hat{\psi }}_{i}(\Phi ,\;E).$$

Algorithm 1 shows the algorithm of our classifier.

## III. METHOD

This section describes how we implemented the classifier explained in Section II.

**Algorithm 1:** The Algorithm of Our Classifier. The Procedure classify(Φ*_k_*, e) Invokes the Feature Extraction Method Φ*_k_* to Classify Signals Recorded by Electrodes e and Output the Selected Target.

**Input:** Signal sets ${\hat{\text{P}}}_{R}(E)$, Feature extraction method Φ, Threshold vector *τ*, The difference between two consecutive window lengths W, Maximum signal length S*_max_*	
**Output:** Classification Output i*,	
1:	N = number of targets
2:	K = number of feature extraction methods
3:	V = K × N zero vector
4:	S = number of samples
5:	*γ* = 0, j = 0
6:	**while** *γ* ≤ *τ*[j] **do**
7:	**for** k in 1 … K **do**
8:	**for** signals set e in ${\hat{\text{P}}}_{R}(E)$ **do**
9:	target = classify(Φ_k_, e)
10:	V[k, target] = V[k, target] + 1
11:	**end for**
12:	**end for**
13:	V = V / R
14:	**for** n in 1 … N **do**
15:	psi[n] = (1/K) × norm(V[:, n], 2)
16:	**end for**
17:	[*γ*, i*] = max(psi)
18:	S = S + W
19:	j = j + 1
20:	**if** S > S*_max_* **then**
21:	break
22:	**end if**
23:	**end while**
24:	**return** i*

### A. Dataset

We use the benchmark dataset for SSVEP-based BCIs [22] for all our experiments. This dataset contains data from 35 healthy participants (S1, S2, … , S35) who use a 40-target SSVEP speller. Each target flashes at a unique frequency *f* ∈ {8.0, 8.2, … , 15.8}Hz and a (non-unique) phase *ϕ* ∈ {0, *π/*2, *π*, 3*π/*2}. Each participant’s data contains 240 (40 × 6) trials, where every target is selected exactly six times. Sixty-four channels of EEG were recorded at a sampling rate of 1000Hz (down-sampled to 250Hz).

### B. Performance Metrics

The information transfer rate (ITR) is the primary measure by which we compared classifiers. ITR formulates the trade-offs among classification delay, window length, and the number of targets. ITR is defined as:

(5) $$C=\frac{60}{T}\times \left[\log_{2}N+P\cdot \log_{2}P+(1-P)\cdot \log_{2}\frac{1-P}{N-1}\right],$$

where *C* is the ITR in bits-per-minute (bpm), *T* is the window-length in seconds, *N* denotes the number of targets, and *P* is the probability of correct classification (with the convention that 0 log 0 = 0). In this work, we include in *T* the 0.5 s pre-stimulation period.

High ITR spellers are of limited practical interest unless they can deliver an acceptable accuracy (typically ≥ 70%). Hence, whenever relevant, this work also compares the performance in terms of accuracy, which is the ratio of the number of correct classifications to the total number of classifications.

### C. Classifiers Implemented for Comparison

Our implementation of MEC, MSI, and FBCCA uses the same parameters as those used by Friman *et al.* [11], Zhang *et al.* [23], and Chen *et al.* [14], respectively (Table I). We, however, extend the second cutoff frequency of the band-pass filter (BPF) for MSI to 50Hz to retain the information of the third harmonic of the highest stimulation frequency (15.8Hz).

### D. Parameter Selection for Our Classifier

For the experiments, our classifier uses three feature extraction methods (MEC, MSI, and FBCCA) and 512 random channel selections (i.e., *R* = 512). The electrode set *E* in Eq. (2) includes 21 electrodes that cover occipital, posterior, and parietal regions of the scalp: P[7, 5, 3, 1, Z, 2, 4, 6, 8], PO[7, 5, 3, z, 4, 6, 8], O[1, z, 2], and CB[1, 2]. All other parameters of feature extraction methods are set per Table I.

We configured the classifier to use 15 window lengths from 0.7 s to 2.1 s in 0.1 s increments, where time 0 denotes the onset of the stimulation. If no target obtains the majority of votes (defined by threshold *τ* ) at 0.7 s, the classifier dynamically increases the window length to 0.8 s. If no target has the majority of votes at 0.8 s, the classifier extends the window-length to 0.9 s and so on. If the window length reaches 2.1 s, the classifier picks the target with the largest number of votes as the classification output, regardless of the value of the threshold at that window length.

For determining the threshold values of each window length, we use the prior assumption that longer window lengths and more data samples improve the classifier’s accuracy. To model this, we choose *t* equidistant thresholds from [*τ*_*min*_, *τ*_*max*_] interval in *descending* order, where *t* is the number of window lengths (15 in our implementation), *τ*_*min*_ ∈ [0, 1], *τ*_*max*_ ∈ [0, 1], and *τ*_*min*_ < *τ*_*max*_. In this modeling, we use *τ*_*min*_ and *τ*_*max*_ for window lengths of 2.1 s and 0.7 s, respectively. The values set for *τ*_*min*_ and *τ*_*max*_ control the behavior of the classifier. Overall, decreasing *τ*_*min*_ and *τ*_*max*_ encourages the algorithm to use shorter window lengths on average. This is a suitable scenario for applications that are tolerant to low accuracies but require high ITR. Alternatively, increasing *τ*_*min*_ and *τ*_*max*_ improves the overall accuracy at the cost of extending the window lengths. Defining *τ*_*min*_ and *τ*_*max*_ as parameters enables users to control the tradeoff between classification speed and classification accuracy. Users can avoid making a design choice on the value of *τ*_*min*_ and *τ*_*max*_ by setting *τ*_*min*_ = *τ*_*max*_ = 0.5, corresponding to a simple 50% majority.

### E. Experimental Setup

We implemented all algorithms on MATLAB 2017a (9.2.0) on a remote host that ran Oracle® Linux Server (Release 7.7). The host was equipped with Intel® Xeon® E5–2680 v4 and 256 GB of memory.

## IV. NUMERICAL RESULTS

In this section, we evaluate the performance of our classifier.

### A. Overall Classifier Performance

**Fig. 1.**
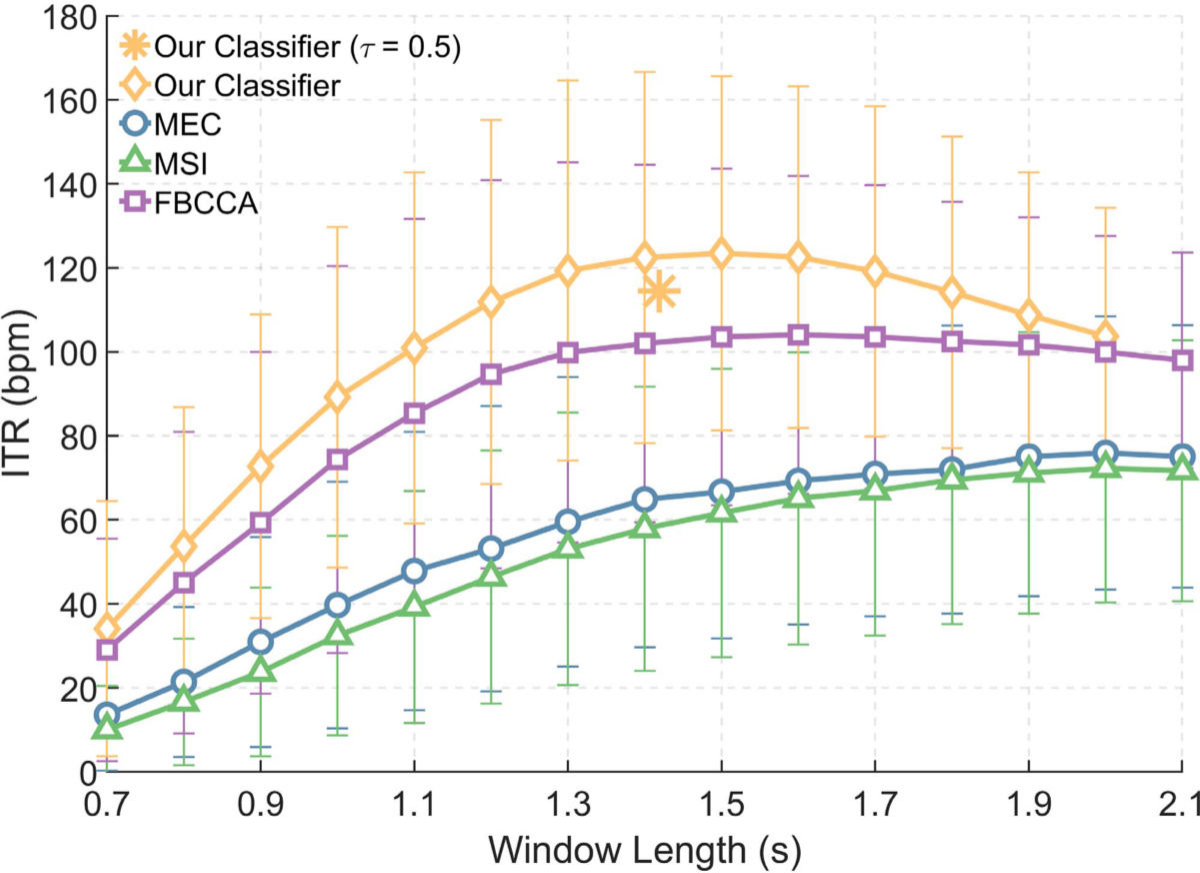
Average ITR of our classifier compared with the average ITR of MEC, MSI, and FBCCA when applied to all 35 participants in our dataset. For our classifier, we set Φ = [MEC, MSI, FBCCA] and used a different value for *τ*_min_ and *τ*_max_ to obtain the average ITR for each average window length. As shown in the figure, we can achieve near-optimal ITR by setting *τ*_min_ = *τ*_max_ = 0.5, a simple majority voting that does not require any prior selection of *τ*_min_ and *τ*_max_. Figure S4 provides a comparison between our classifier and CCA.

Figure 1 depicts the average ITR of our classifier (See Section II) for different window lengths and compares it with MEC [11], MSI [23], and FBCCA [14]. MEC gives a maximum ITR of 75.9bpm at the fixed window length of 2.0 s corresponding to an accuracy of 72.0%. MSI and FBCCA yield a maximum ITR of 72.2bpm and 104.0bpm for window lengths of 2.0 s and 1.6 s and corresponding accuracies of 69.8% and 79.1%, respectively. When compared to MEC, MSI, and FBCCA, our classifier increased the maximum average ITR by 47.5bpm (*p* ≤ 0.01), 51.2bpm (*p* ≤ 0.01), and 19.5bpm (*p* ≤ 0.01), respectively.

Our classifier uses dynamic window lengths; different pairs of *τ*_*min*_ and *τ*_*max*_ result in different average window lengths. In Fig. 1, we classified the dataset using numerous values for *τ*_*min*_ and *τ*_*max*_ to obtain at least one average ITR for each average window length. We then selected the maximum ITR at each average window length to obtain the final result. Determining *τ*_*min*_ and *τ*_*max*_, however, adds a new design choice to the classifier. To avoid making a decision on *τ*_*min*_ and *τ*_*max*_, we can set *τ*_*min*_ = *τ*_*max*_ = 0.5, corresponding to a simple majority vote. Figure 1 shows that for *τ*_*min*_ = *τ*_*max*_ = 0.5, the classifier had an average accuracy of 81.6% at the average window length of 1.5 s, corresponding to an average ITR of 114.5bpm. Figures S6–S40 show the classifier’s performance for individual participants.

### B. Feature Selection Performance

**Fig. 2.**
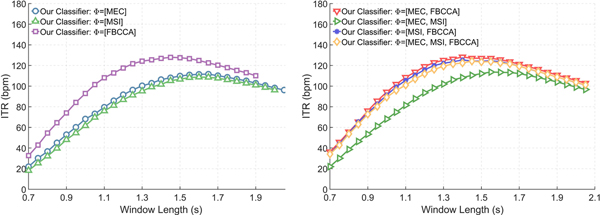
The average ITR of our classifier—with dynamic channel selection and dynamic window length—configured to use (left) three individual feature extraction methods and (right) four different combinations of feature extraction methods.

Figure 2 compares our classifier’s performance for configurations that use single (left) and multiple (right) feature extraction methods. For configurations with a single feature extraction method, our classifier increased the maximum average ITR to 111.6bpm, 108.8bpm, and 127.9bpm for configurations with Φ = [MEC], Φ = [MSI], and = [FBCCA], respectively (*p* ≤ 0.01). Among the configurations that used multiple feature extraction methods, the configuration that used Φ = [MEC, FBCCA] had the largest average ITR at 128.2bpm.

### C. Dynamic Channel Selection Performance

**Fig. 3.**
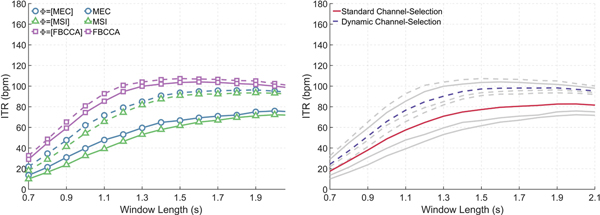
(left) The average ITR for three feature extraction methods MEC, MSI, and FBCCA with (dashed line) and without (solid lines) the dynamic channel-selection method discussed in Section II–B. (right) The ITR of all three feature extraction methods (MEC, MSI, and FBCCA) that use dynamic (dashed) and standard channel selection averaged into a single curve (Results from the left plot (greyed-out lines) and are added here for comparison).

Figure 3 (left) compares the average ITR of MEC, MSI, and FBCCA with our classifier (for three configurations, where (i) Φ = [MEC], (ii) Φ = [MSI], and (iii) Φ = [FBCCA].) To evaluate the performance of the proposed channel selection, the classifier used fixed window lengths. The proposed channel selection increased the maximum ITR from 75.9 to 96.3bpm for MEC, from 72.2 to 93.4bpm for MSI (*p* ≤ 0.01) but the changes for FBCCA were not significant.

Figure 3 (right) averages the ITR of MEC, MSI and FBCCA and compares it with the average ITR across the three configurations of our classifier. It shows that on average, the dynamic channel selection increases the maximum ITR from 82.7bpm (at the corresponding window length of 2.0 s) to 98.3bpm (*p* ≤ 0.01).

### D. Dynamic Window Length Performance

Table II details the classification performance for each permissible window length. The results are obtained for Φ = [MEC, MSI, FBCCA] $\tau_{min}^{*}=0.135$ and $\tau_{max}^{*}=0.865$. The very high accuracy associated with window lengths shorter than ≤ 2.1 s confirms the efficacy of the proposed features; 73.02% of the dataset can be classified with the accuracy of 90.25% with the average window length of 1.30 s. Around 4.68% of the signals (corresponding to 393 signals) are classified at the window length of 2.1 s with the accuracy of 45.04%. The continuous decrease in overall accuracy for larger window lengths is expected because the algorithm defers the classification of only noisy signals to these window lengths.

## V. DISCUSSION AND FUTURE WORK

Our classifier has several significant advantages over existing SSVEP classifiers. First, because it uses multiple feature extraction methods, it does not depend on the specious assumption that one feature extraction method will consistently outperform the others. Second, because this classifier uses many channel selections, there is no need to pick a specific channel selection for a specific user or specific application. Third, the classifier adjusts the window length for each classification. Together, these three properties improve the average ITR. Fourth, because it automates choices about feature extraction, channel selection, and window length, the classifier is easy for caregivers and others to use; it does not require special expertise. The rest of this section discusses other advantages of this classifier and opportunities for improving it.

The classifier uses voting to combine the features extracted by the different methods. While conventional normalization techniques can rescale and combine features (e.g., using the logistic function to convert features to probabilities), these techniques often lead to loss of *interpretability* because the normalized features represent disparate phenomena (e.g., even after normalization, combining correlation with SNR is difficult). On the other hand, using voting to combine features satisfies many *desiderata* of interpretability including transparency (i.e., it is clear how the classifier works), trustworthiness (i.e., confidence that the classifier performs well), and transferability (i.e., classifier can function in environments different from the test environment) [24].

The parameter *ψ*_*i*_(Φ, *E*), as computed per Eq. (1), is the probability that a random selection of a channel set and a feature extraction method result in a vote for target *i*. As *ψ*_*i*_(Φ, *E*) approaches one, all possible selections lead to the same result. In these cases, it becomes less important to pick one selection over the others. Thus, instead of searching for the best selection of channel set and feature extraction method, the classifier dynamically increases the window length until all (or most of) the selections result in the same output. For a non-target *i* (i.e., any target other than the one the user intends to selects), it is unlikely (although not impossible) to obtain *ψ*_*i*_(Φ, *E*) = 1. This is because non-targets generally have smaller SNRs, which makes the classification results more random. This is confirmed by the results provided in Table II, where signals with shorter window lengths (and higher *ψ*_*i*_) are classified with an average accuracy of more than 90%.

Brain injuries, aging, and other neuroplasticity can change the spatial distribution of SSVEPs [25]–[27]. Hence, a fixed channel selection can limit the system’s applicability. The uniform selection of random electrode sets, as explained in Section II, mitigates this problem [28]. Inherent symmetries of the uniform distribution allow unbiased selection of different spatial distributions. Thus, SSVEP detection becomes independent of their spatial distribution.

The advantages of our proposed channel-selection technique are obtained at no cost to the classifier’s performance. To confirm this, we configured MEC, MSI, and FBCCA to use our technique (results in Fig. 3). The technique significantly improved the average ITR for MEC and MSI, but not for FBCCA (for most window lengths). We attribute this at least in part to the fact that, unlike MEC and MSI parameters, FBCCA’s parameters (Table I) were already optimized for our dataset (or at least a subset of our dataset). Hence, Fig. 3 implies that our classifier mitigates the deleterious impact of lack of training and parameter optimization. If the parameters are already optimized, our classifier does not impair classification. One potential way to improve the performance of our classifier is to test the inclusion of different feature extraction methods. Possible choices include filter bank MEC [29], deep multi-set CCA (DMCCA) [30], and task-related component analysis (TRCA) [31].

Our classifier is computationally complex. The computational complexity of the classifier depends on *K R*, where *K* is the number of feature extraction methods in Φ and *R* is the number of channel selections. In our implementation, *K* = 3 and *R* = 512. Thus, our classifier is roughly 1536 times more computationally complex than a classifier with *K* = 1 and *R* = 1. There are a number of ways to mitigate this added complexity. First, as Fig. S2 shows, using *R* = 50 results in similar performance to *R* = 512. This simple change reduces the computational complexity of our classifier by a factor of ten. In addition, our classifier is highly parallelizable—every vote can be computed simultaneously. We developed a graphics processing unit (GPU)-accelerated version of our classifier to demonstrate its parallelizability. As shown in Fig. S3, the run time of the GPU-accelerated version was 0.05 s, 230× faster than the MATLAB version of our classifier (11.52 s).

Different sampling strategies might improve the channel-selection. Let *r* be the cardinality of a random subset of P(E), where *E* includes the 21 electrodes discussed in Section III–D and all subsets are equally probable. Then, *r* approximately follows a normal distribution $r\sim N(\mu =10.5,\;\sigma^{2}=\;\text{5}.\text{25})\;$. One possible improvement is to change the expected number of channels (*μ*) by changing window length. This is based on the observation that the number of useful channels usually increases with window length, presumably because more data reduces noise. Another possible improvement is using a non-uniform spatial distribution to select electrodes (instead of a uniform distribution). This could increase the probability of selecting certain electrodes (e.g., Oz).

Our classifier works better for some participants than for others. Table II shows that classification accuracy was much lower at longer window lengths (45.04% at 2.1 s) than it was at shorter window lengths (94.17% at 1.2 s). The majority of the signals (80.9% (See Table III)) classified at a window length of 2.1 s came from just nine of the 35 participants. Thus, window length might identify people for whom an alternative classification strategy might perform better.

Rather than increasing window length, the classifier could use other methods to address low classification confidence. For example, it could re-assign flashing frequency and target phase to distinguish among the most probable targets (e.g., switching to hierarchical selection only when necessary). In the trade-off between classification accuracy and latency, a conservative (i.e., high) threshold biases toward accuracy. This benefits applications that have low tolerance for error (e.g., wheelchair control).

## VI. CONCLUSION

We propose a new dynamic window length SSVEP classifier that uses multiple feature extraction methods and channel selections. Because it automatically selects the feature extraction method and recording channels for each individual and each application, the classifier should be easy for caregivers and others to use.

The classifier evaluates all permutations of different feature extraction methods and channel selections, and it uses voting by the permutations to identify the person’s target. The classifier dynamically extends the window length until either the number of votes for one target exceeds a pre-determined threshold, or the window length reaches a preset maximum value (at which point the target with the most votes is identified).

This classifier has four advantages over commonly used classifiers (i.e., minimum energy combination (MEC), maximum synchronization index (MSI), filter bank canonical correlation coefficient (FBCCA)). First, it does not assume that a single feature extraction method is best. Second, it adapts channel selection to the person and the application. Third, it uses dynamic window lengths. Fourth, it does not require training for feature extraction or channel selection. For 35 participants, the classifier gave an average ITR of 124.1bpm versus 104.0bpm for the next-best classifier (FBCCA).

## Supplementary Material

supp1-3106876

## Figures and Tables

**TABLE I:** Feature Extraction Parameters. Refer to the Cited References for the Definition of Each Parameter

MEC	MSI	FBCCA

N_h_ = 3 / AR Order = 15 / P_3_, O_1_, P_2_, O_Z_, P_4_, O_2_	N_h_ = 3 / *τ* = 1 / BPF: [0.5, 50] Hz* / P_3_, P_2_, P_4_, PO_7_, PO_8_, O_1_, O_Z_, O_2_	N_h_ = 5 / a = 1.25, b = 0.25 / BPF: [*i* × 8, 88] Hz *i* ∈ {1, 2, …, 7} / P_z_, PO_5_, PO_3_, PO_2_, PO_4_, PO_6_ O_1_, O_Z_, O_2_

**TABLE II:** The Relative Population of Signals Classified at Each Window Length and Their Corresponding Classification Accuracy

Window Length (s)	Population (%)	Accuracy (%)

0.7	0.02	50.00
0.8	0.43	91.67
0.9	2.31	94.33
1.0	5.87	92.70
1.1	9.30	93.60
1.2	11.64	94.17
1.3	12.44	91.58
1.4	11.60	89.12
1.5	10.77	86.74
1.6	8.64	82.64
1.7	7.15	75.37
1.8	6.32	69.87
1.9	5.34	66.15
2.0	3.47	62.33
2.1	4.68	45.04

**Mean:** 1.45	**Average:** 6.66	**Mean:** 83.53

**TABLE III:** Performance Summary of All Participants With the Average ITR of Below 100 bpm

Participant	ITR (bpm)	Relative Share (%)	Accuracy @ 2.1s (%)

S11	28.3	25.7	37.6
S29	45.8	9.4	54.1
S16	49.2	3.8	40.0
S21	54.4	0.8	66.7
S33	55.7	16.5	30.8
S7	71.7	1.8	14.3
S19	80.0	16.5	60.0
S8	86.9	3.6	57.1
S18	95.2	2.8	36.4

**Summary**	**Average:** 63.1	**Total:** 80.9	**Average:** 43.4
