# Supplementary material for: A Voting-Enhanced Dynamic-Window-Length Classifier for SSVEP-Based BCIs
Source: IEEE Trans Neural Syst Rehabil Eng. Author manuscript; Available in PMC 2021 Oct 7. (PMC8496754; doi:10.1109/TNSRE.2021.3106876)
Supplement: supp1-3106876 [file NIHMS1738619-supplement-supp1-3106876.pdf]

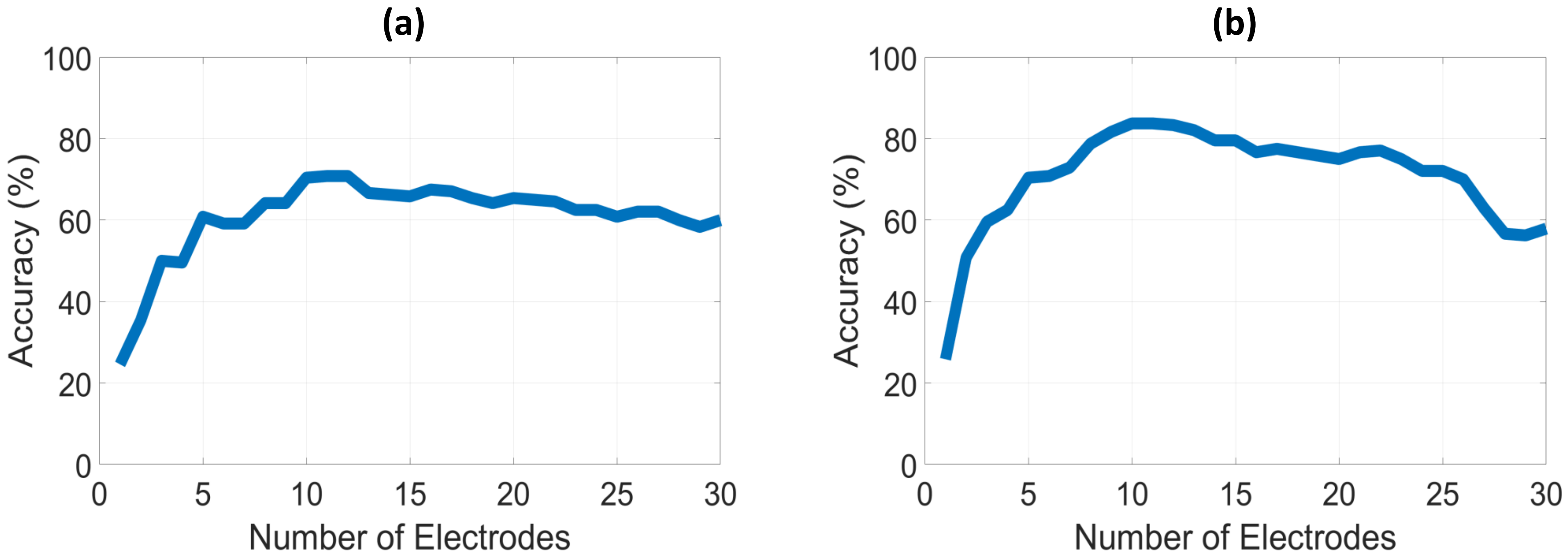

Fig. S1. Average accuracy per different selections of channels for two different subjects and a fixed window-length of 1.5s. In this experiment, the classification was repeated for 29 different selections of channels using FBCCA for feature extraction. The first channel-selection included electrodes 64 and 63. The second selection included 64, 63, and 62. And so on. (a) S1 (b) S2

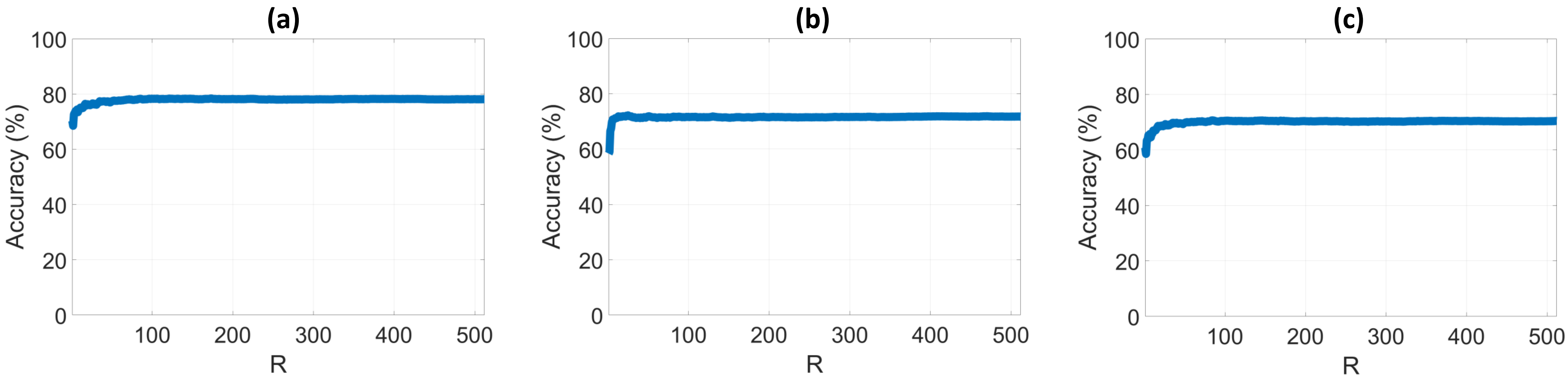

Fig. S2. Average accuracy for all 35 subjects for different values of R for a fixed window-length of 1.5s. (a) FBCCA, (b) MEC, (c) MSI. The average accuracy stabilizes for all three feature extraction methods when 'R' exceeds 100.

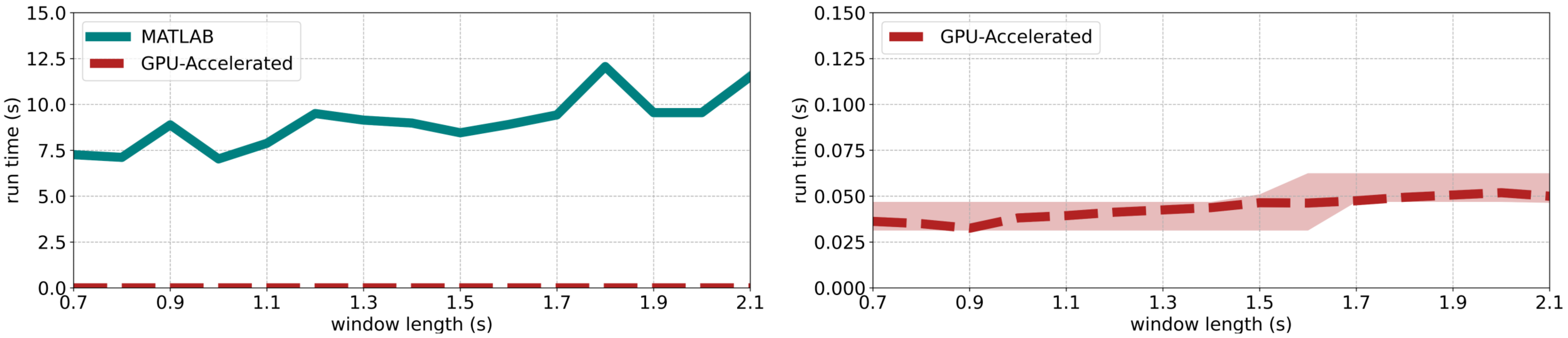

Fig. S3. Run times for the MATLAB-based (solid line in the left figure) and GPU-accelerated (dashed line in the left and right figures) implementation of our classifier. Our classifier was configured to use 64 channel selections and a single feature extraction method (i.e., MEC). The first signal of the first subject was used for benchmarking. The shaded areas represent the slowest and fasted run times (among 25 runs), while the solid lines represent the average run times. The `synchronize()` command was used to ensure accurate run time measurement. The test machine had Microsoft Windows 10, an Intel Core i7-10700K CPU, 32GB of memory, and a NVidia GeForce RTX 2070 Super.

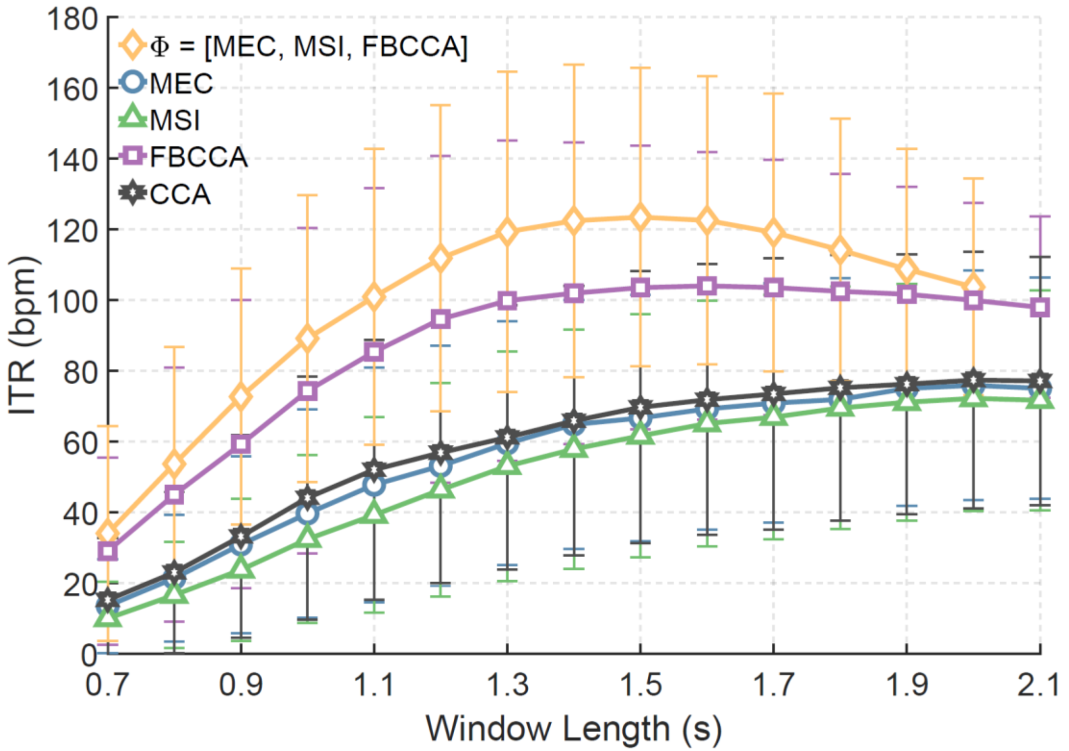

Fig. S4. Average ITR per window length for our proposed classifier and four other commonly used SSVEP classifiers. We used the settings shown in Table I for MEC, MSI, and FBCCA. For CCA, we set the number of harmonics in the template signal to three and used eight electrodes in the occipital and parietal regions of the scalp.

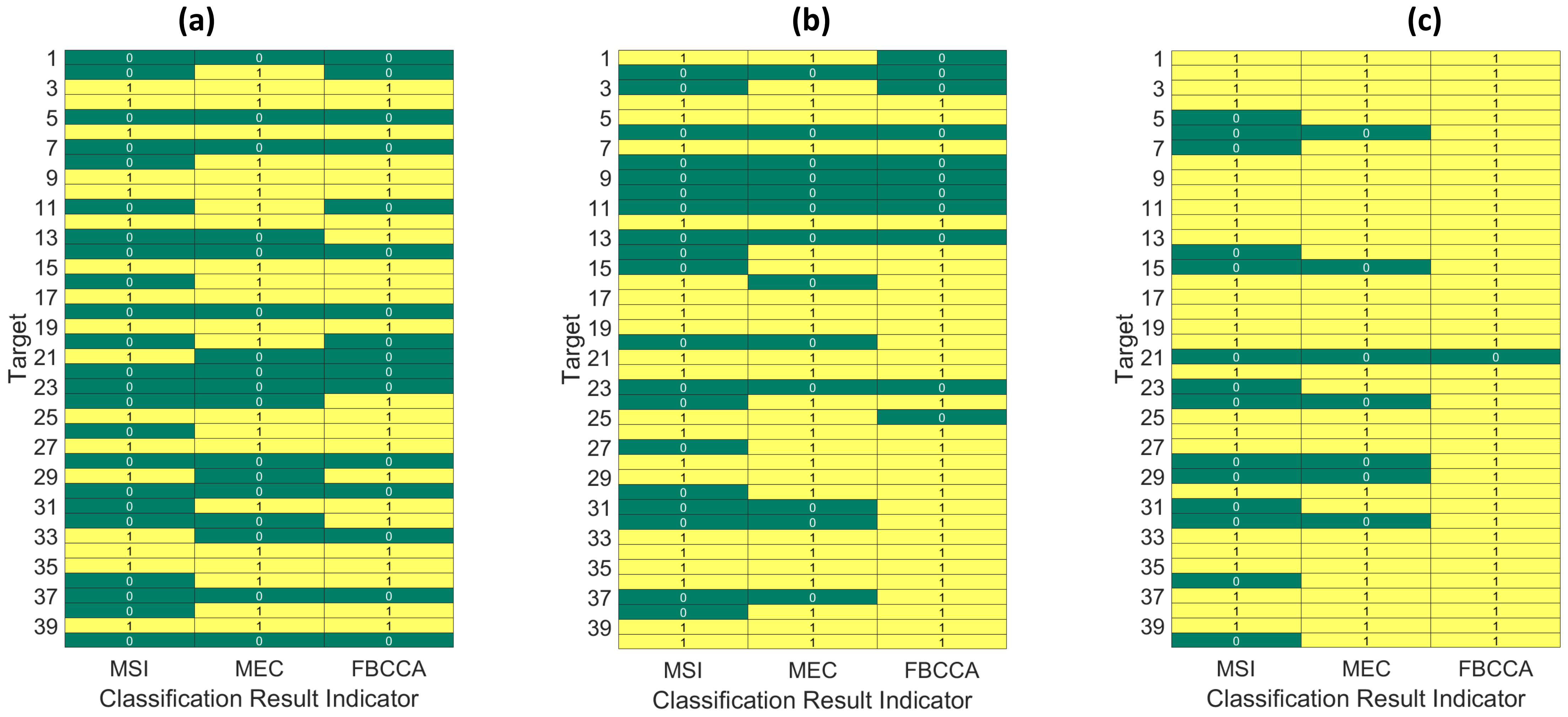

Fig. S5. Classification results using three different feature extraction methods for three participants. The results pertain to the first block for the fixed window length of 1.5s. ‘1’ indicates that the classification was correct while ‘0’ indicates a wrong classification. (a) S1, (b) S2, (c) S3

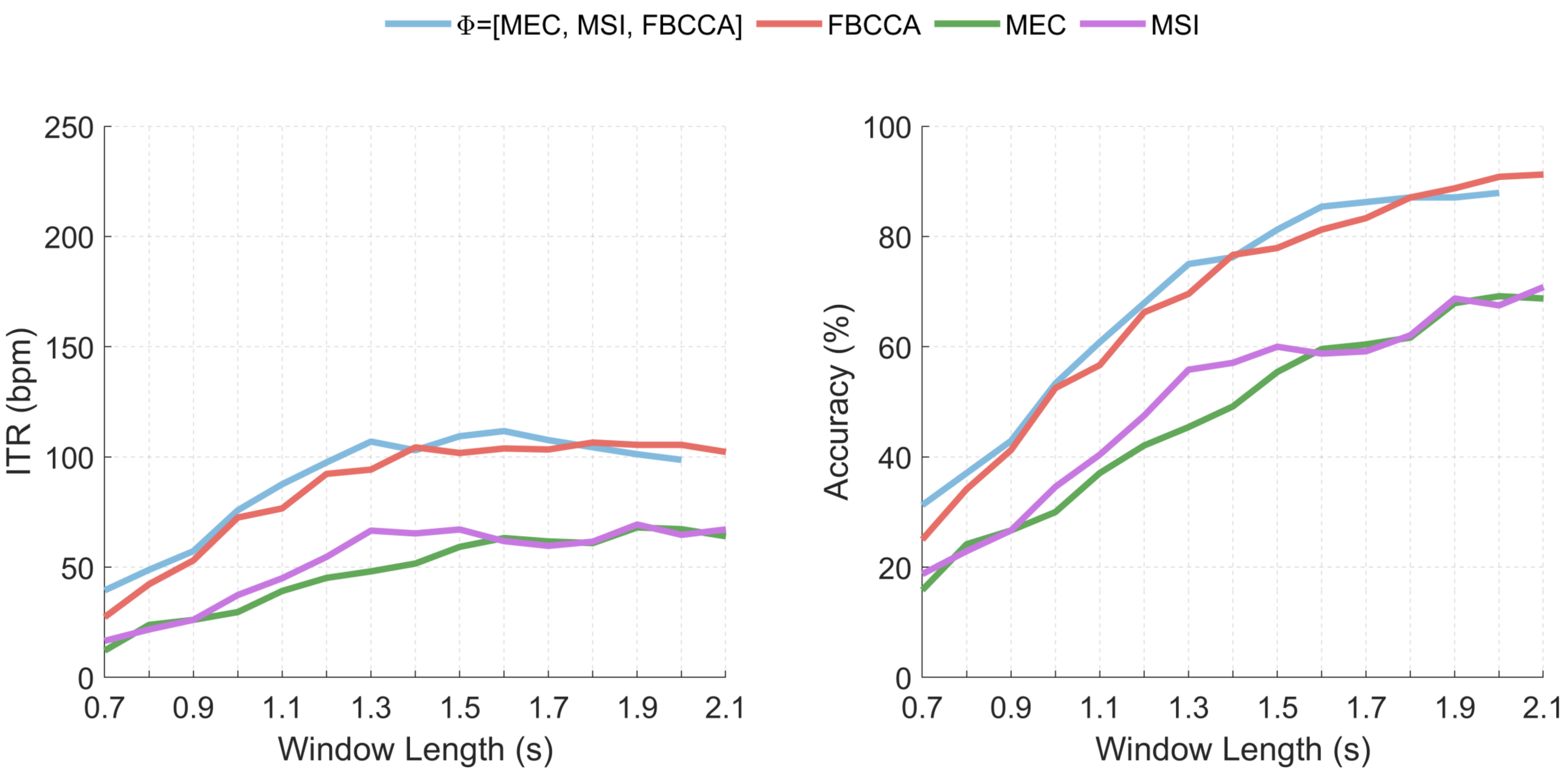

Fig. S6. (left) ITR and (right) classification accuracy for S1

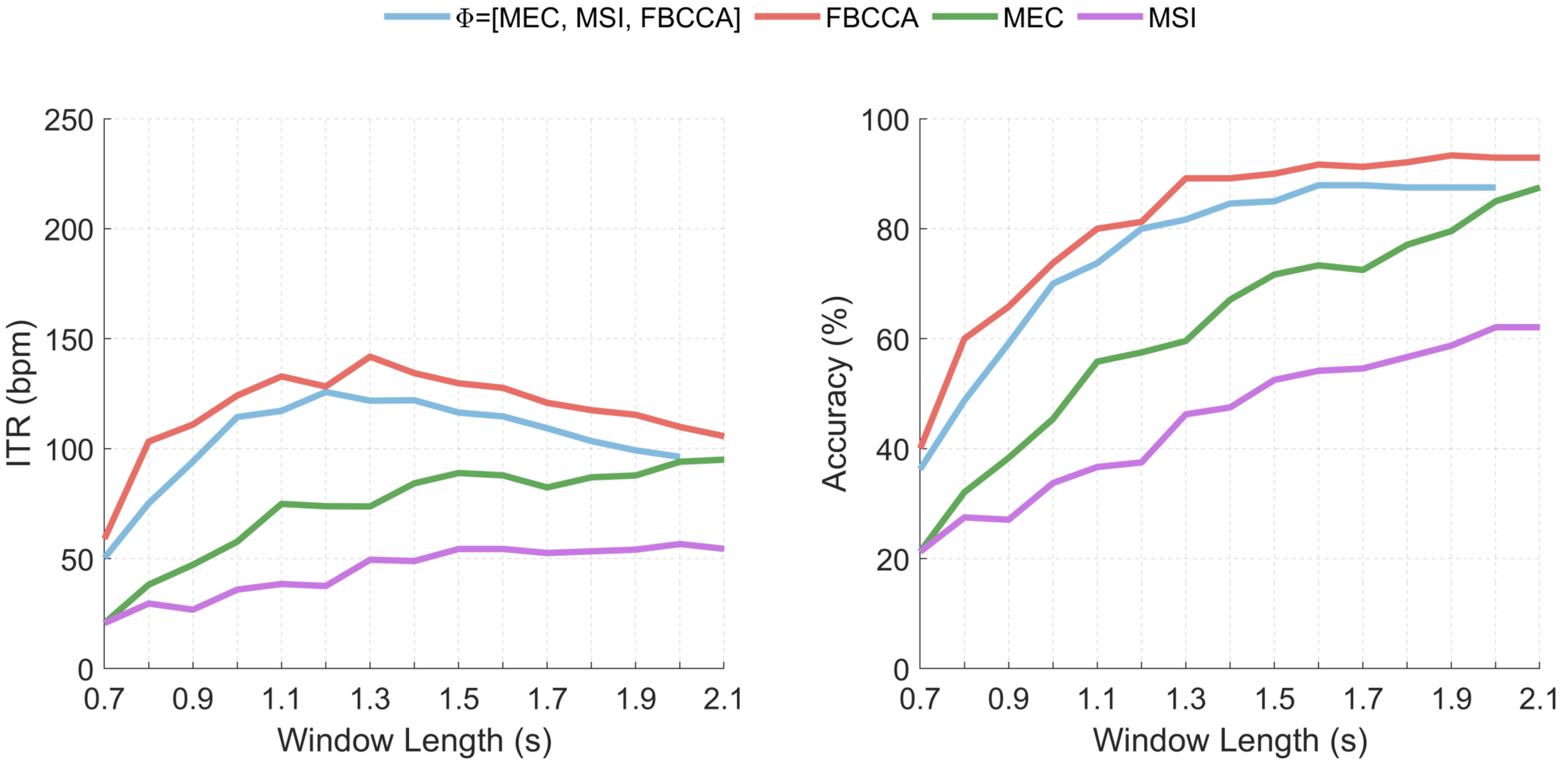

Fig. S7. (left) ITR and (right) classification accuracy for S2

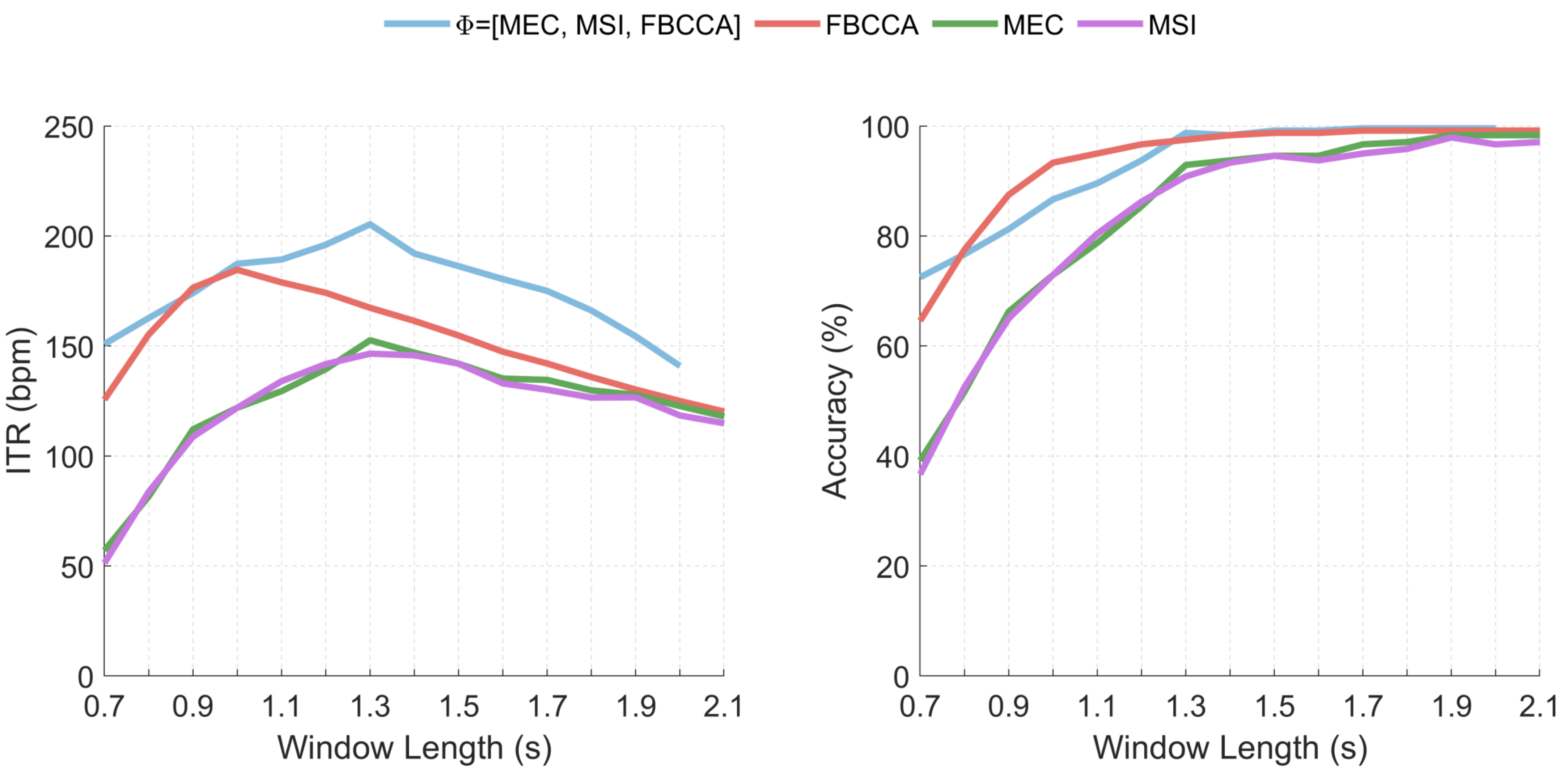

Fig. S8. (left) ITR and (right) classification accuracy for S3

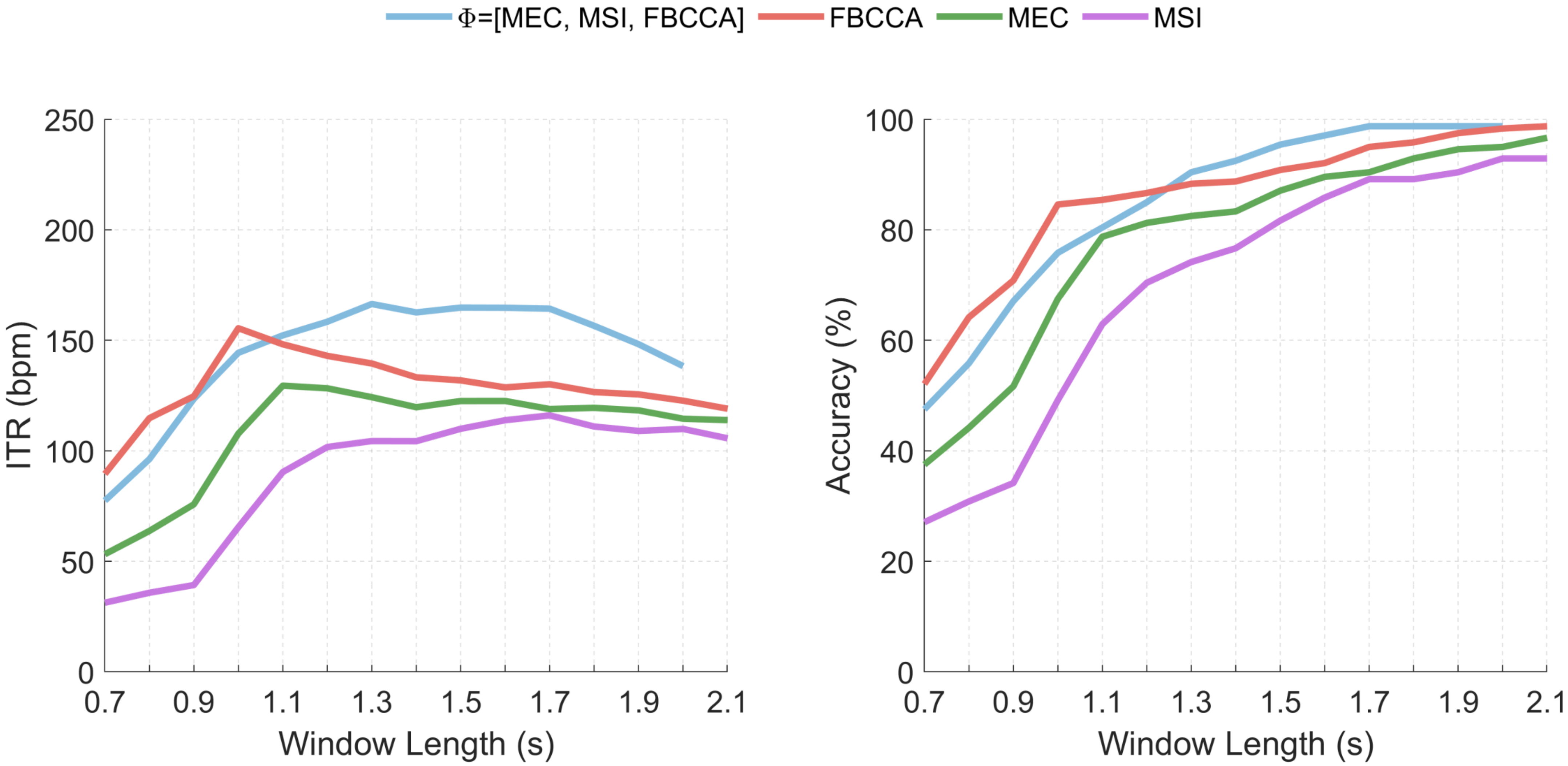

Fig. S9. (left) ITR and (right) classification accuracy for S4

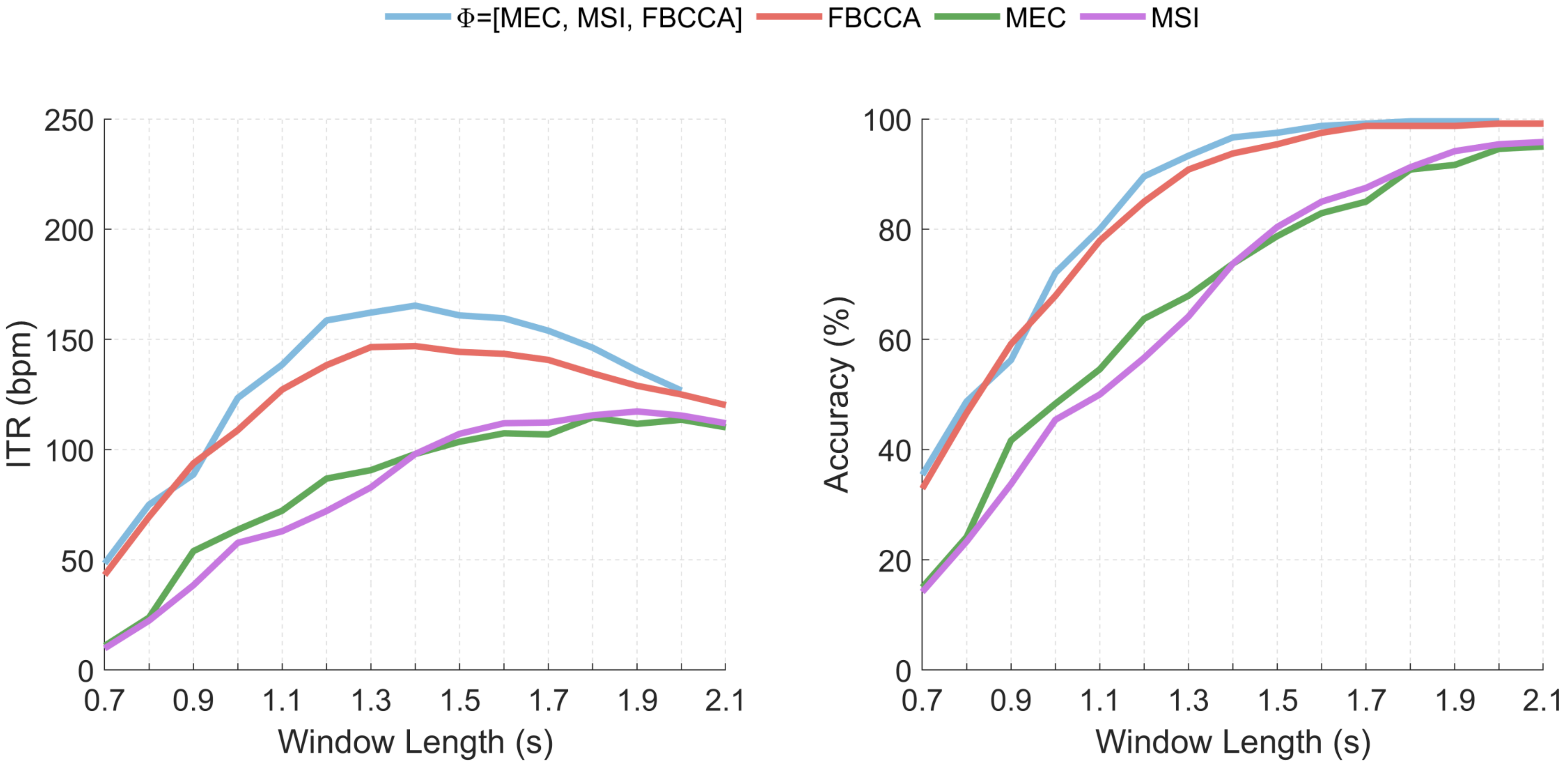

Fig. S10. (left) ITR and (right) classification accuracy for S5

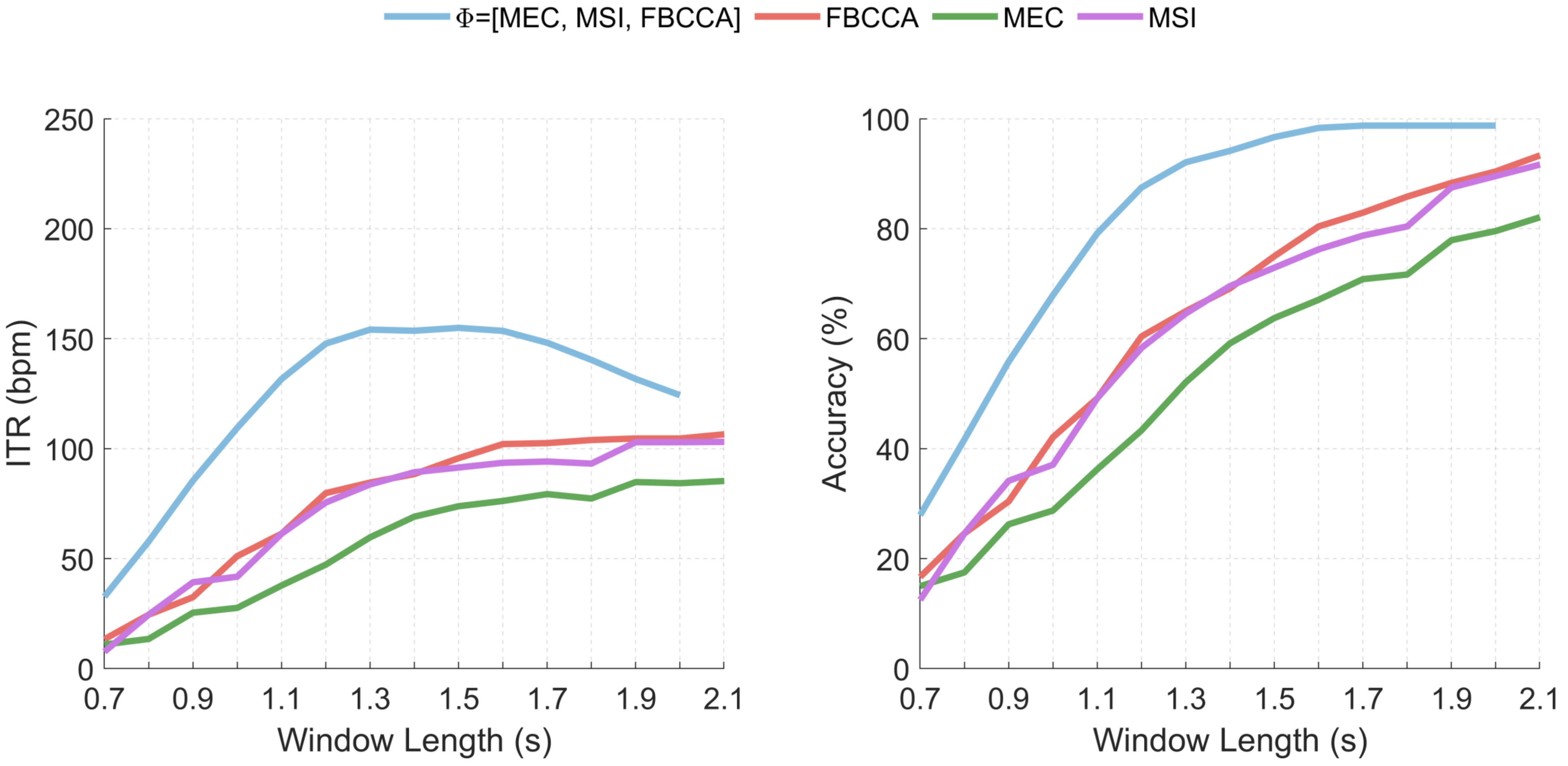

Fig. S11. (left) ITR and (right) classification accuracy for S6

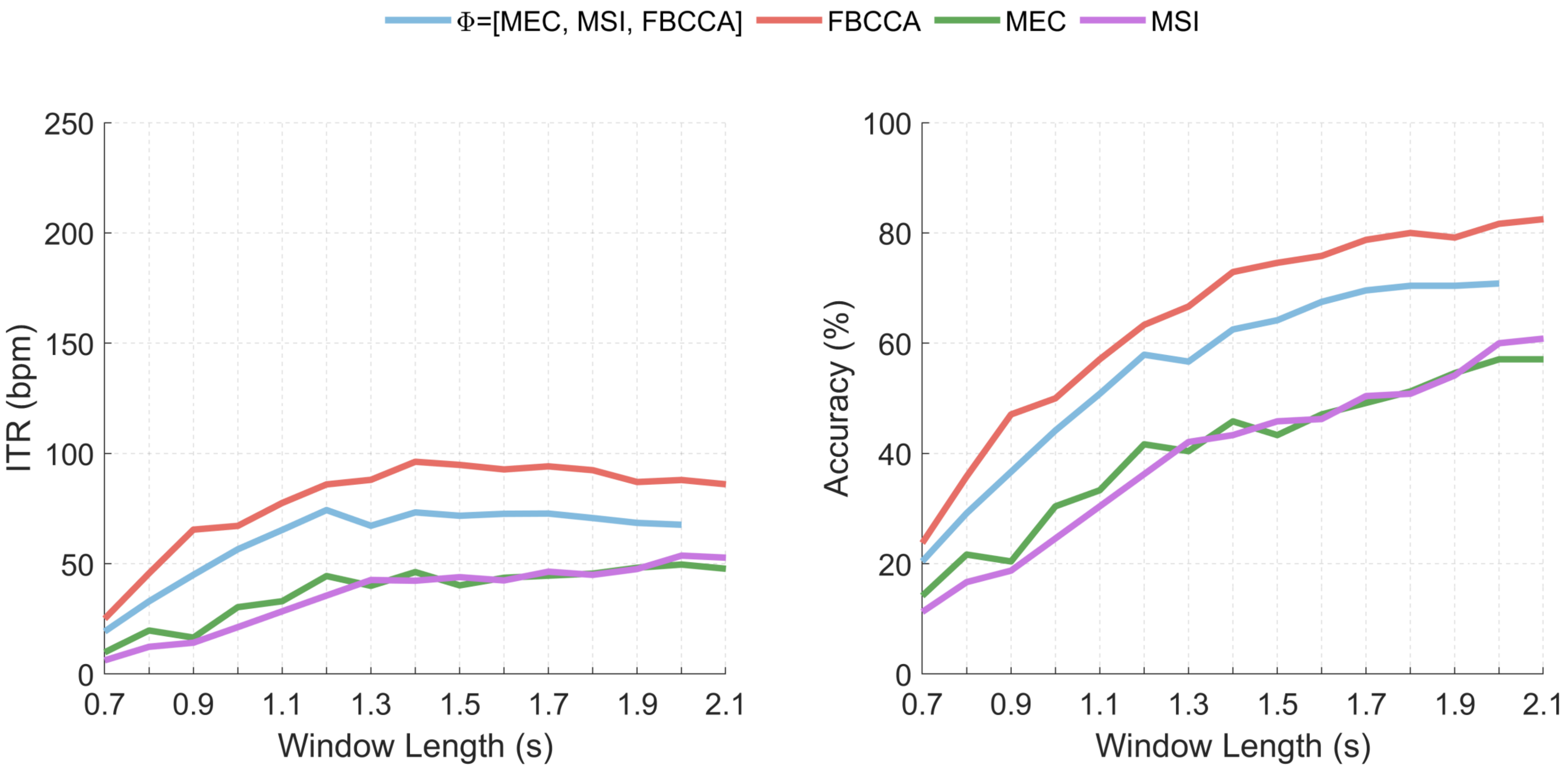

Fig. S12. (left) ITR and (right) classification accuracy for S7

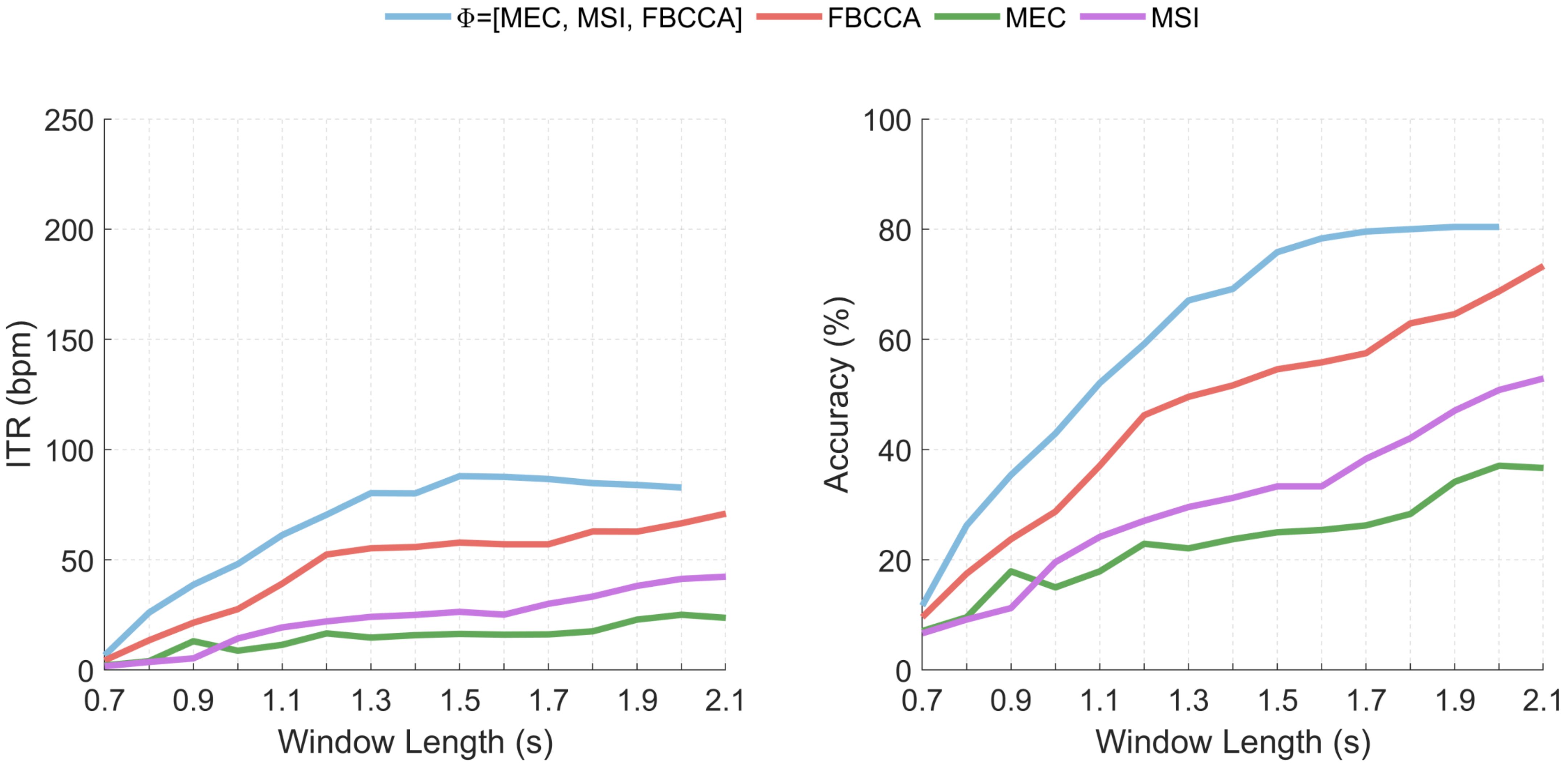

Fig. S13. (left) ITR and (right) classification accuracy for S8

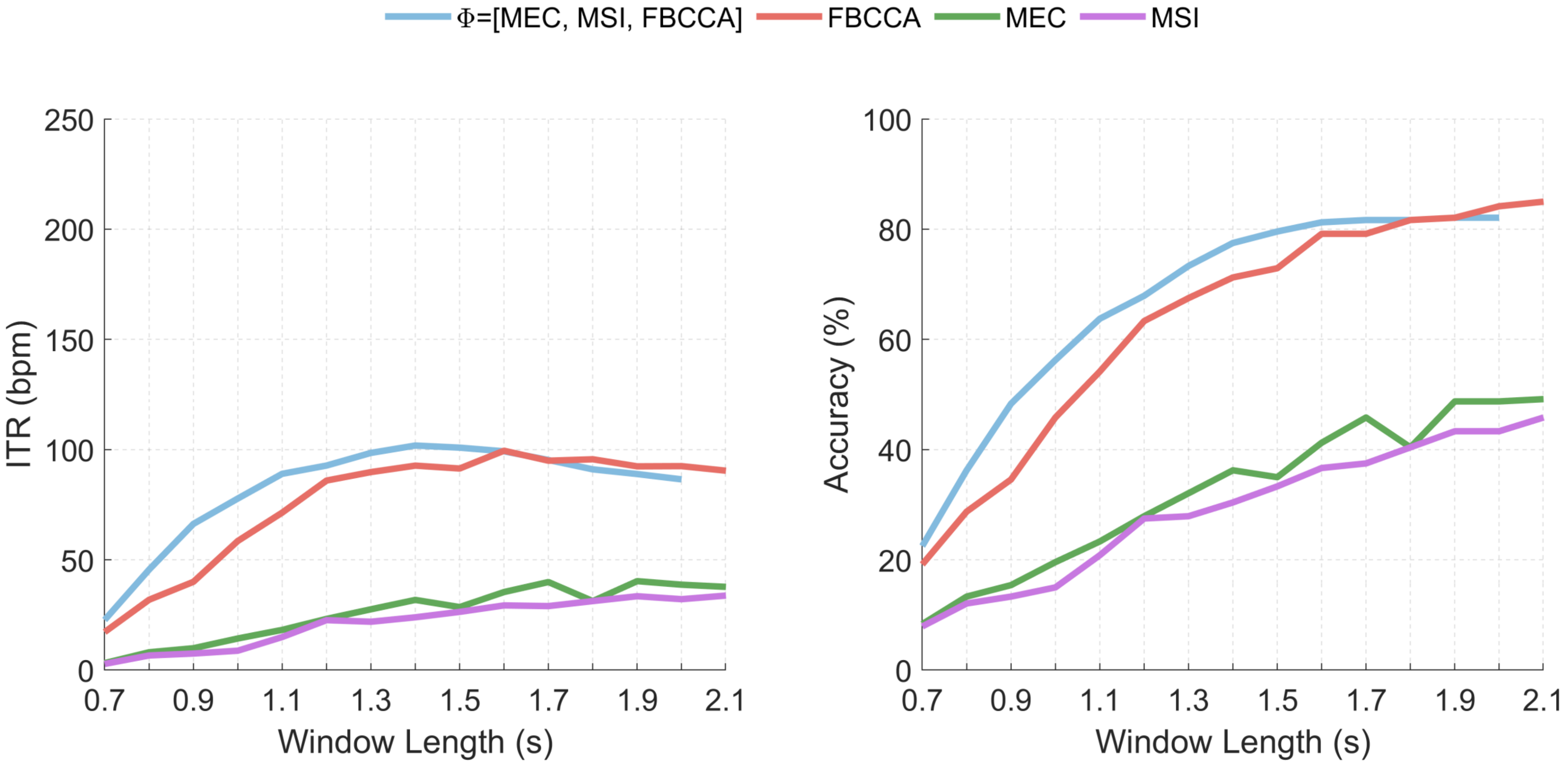

Fig. S14. (left) ITR and (right) classification accuracy for S9

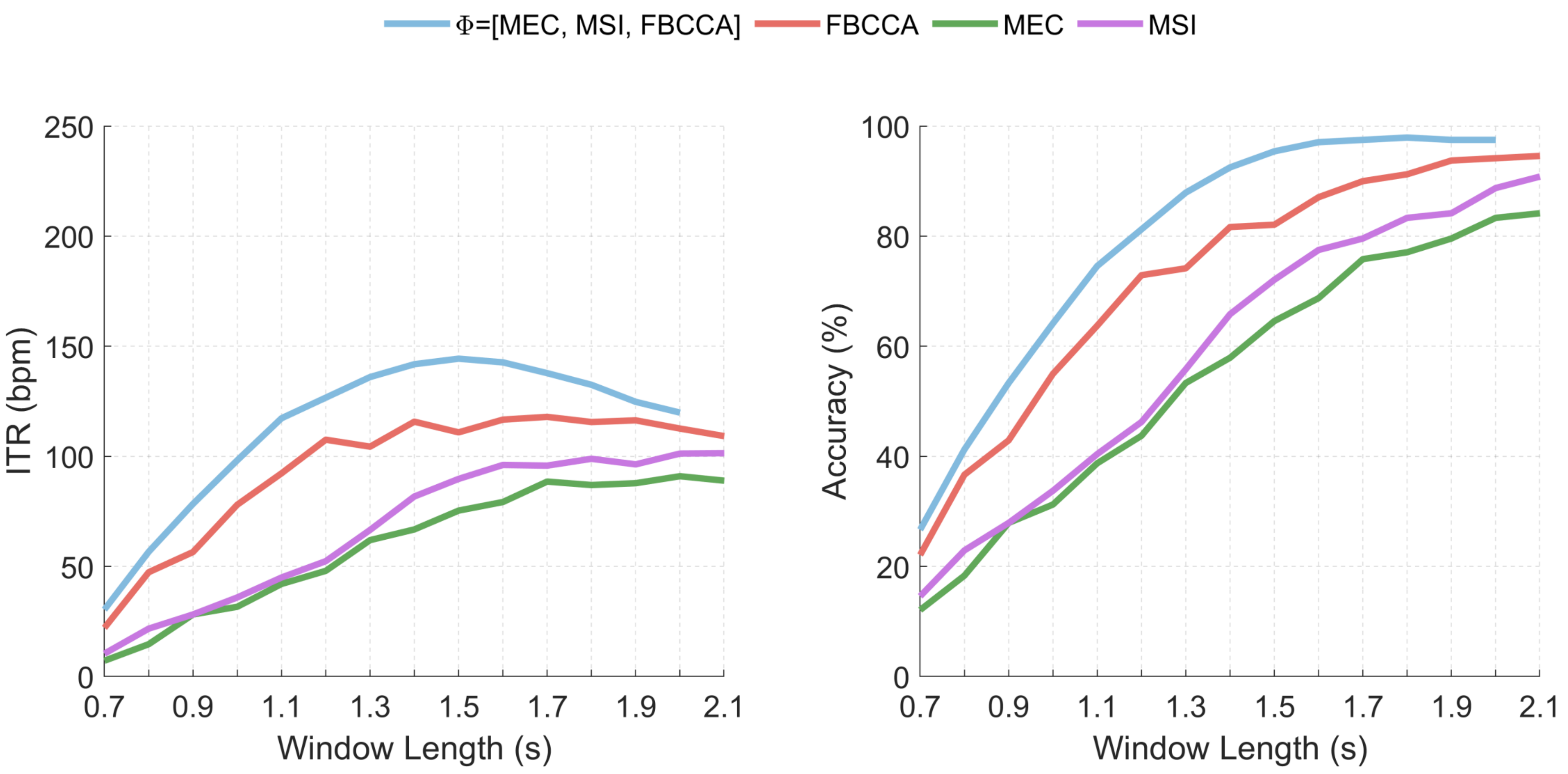

Fig. S15. (left) ITR and (right) classification accuracy for S10

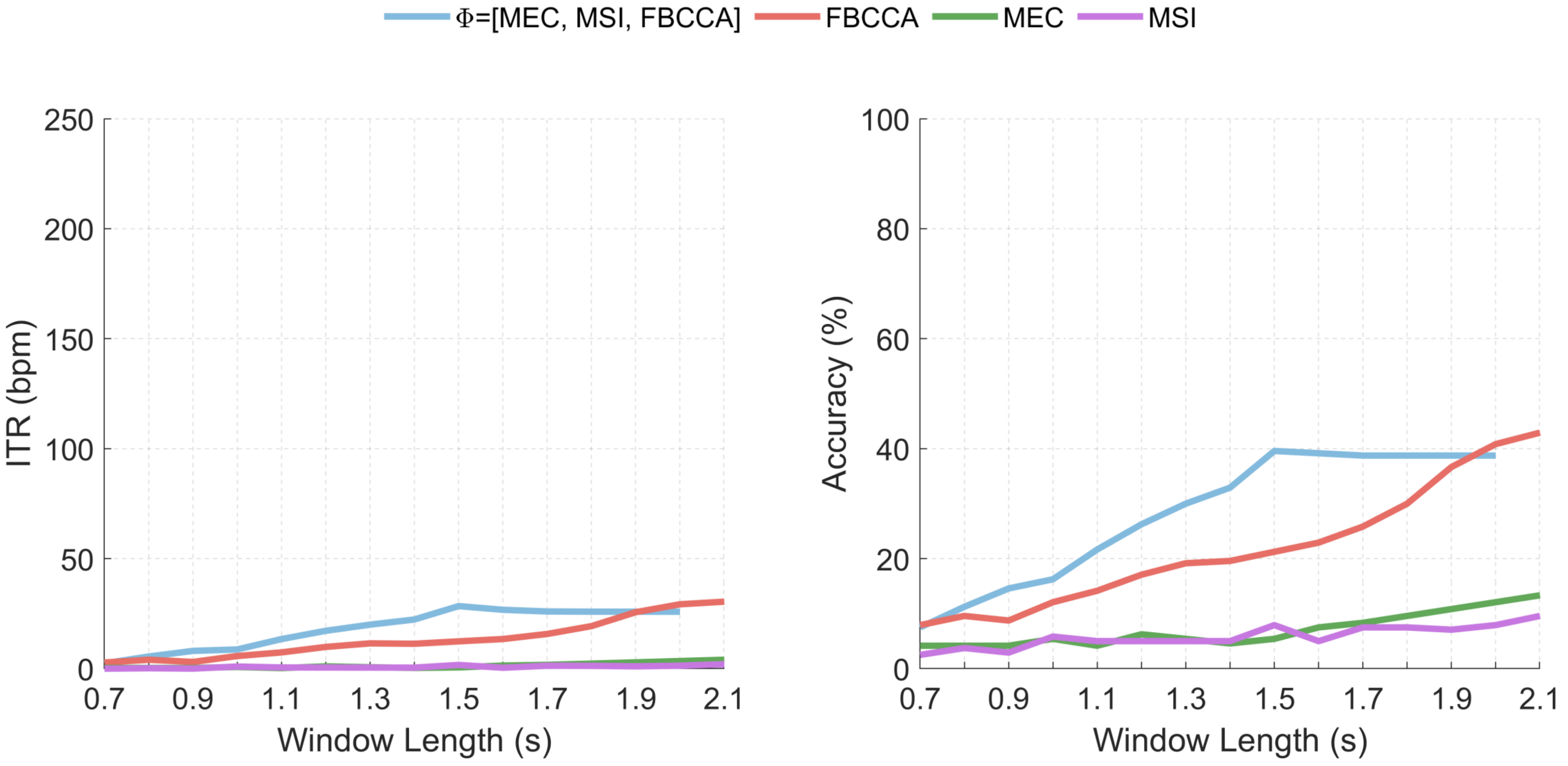

Fig. S16. (left) ITR and (right) classification accuracy for S11

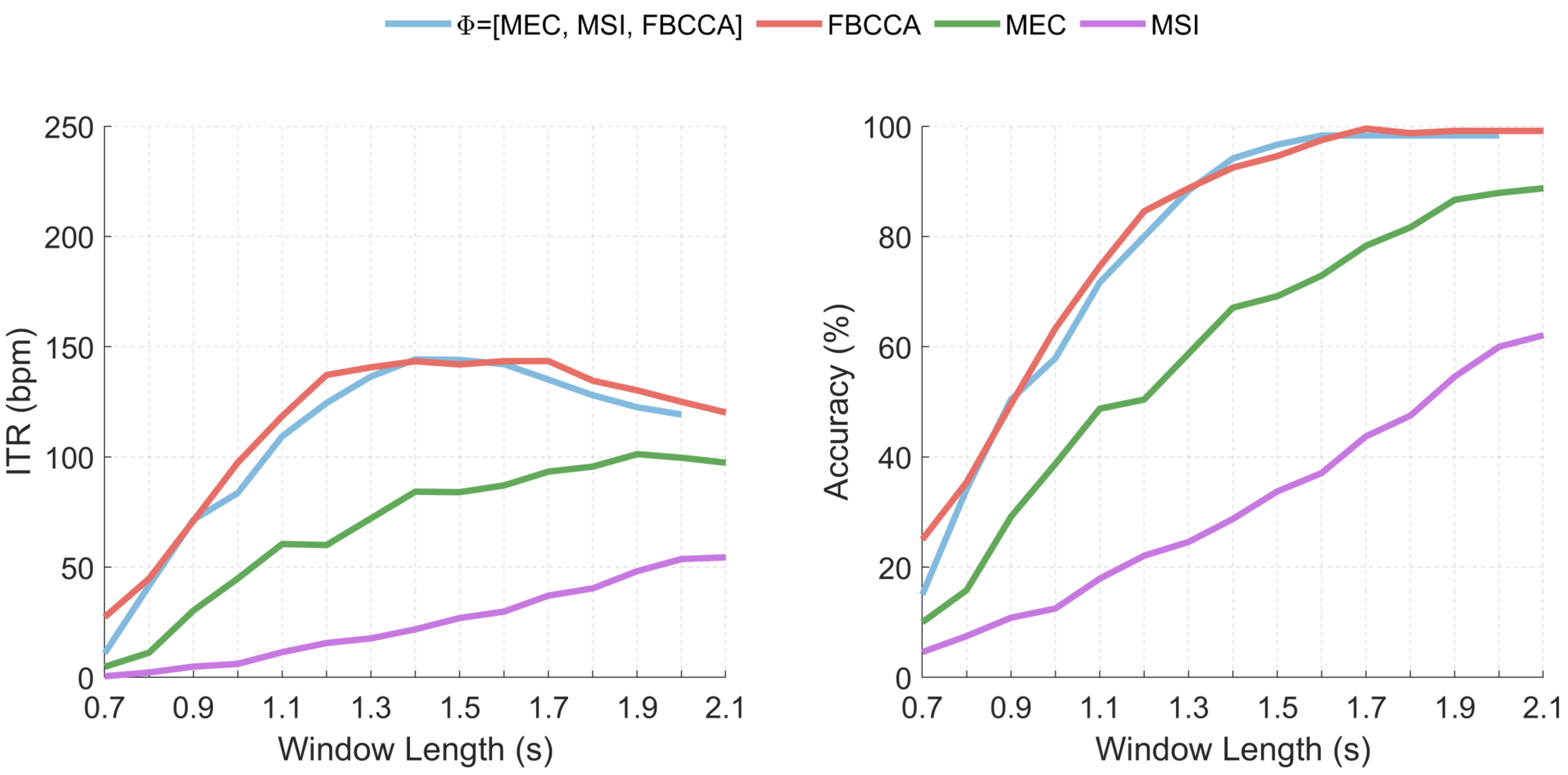

Fig. S17. (left) ITR and (right) classification accuracy for S12

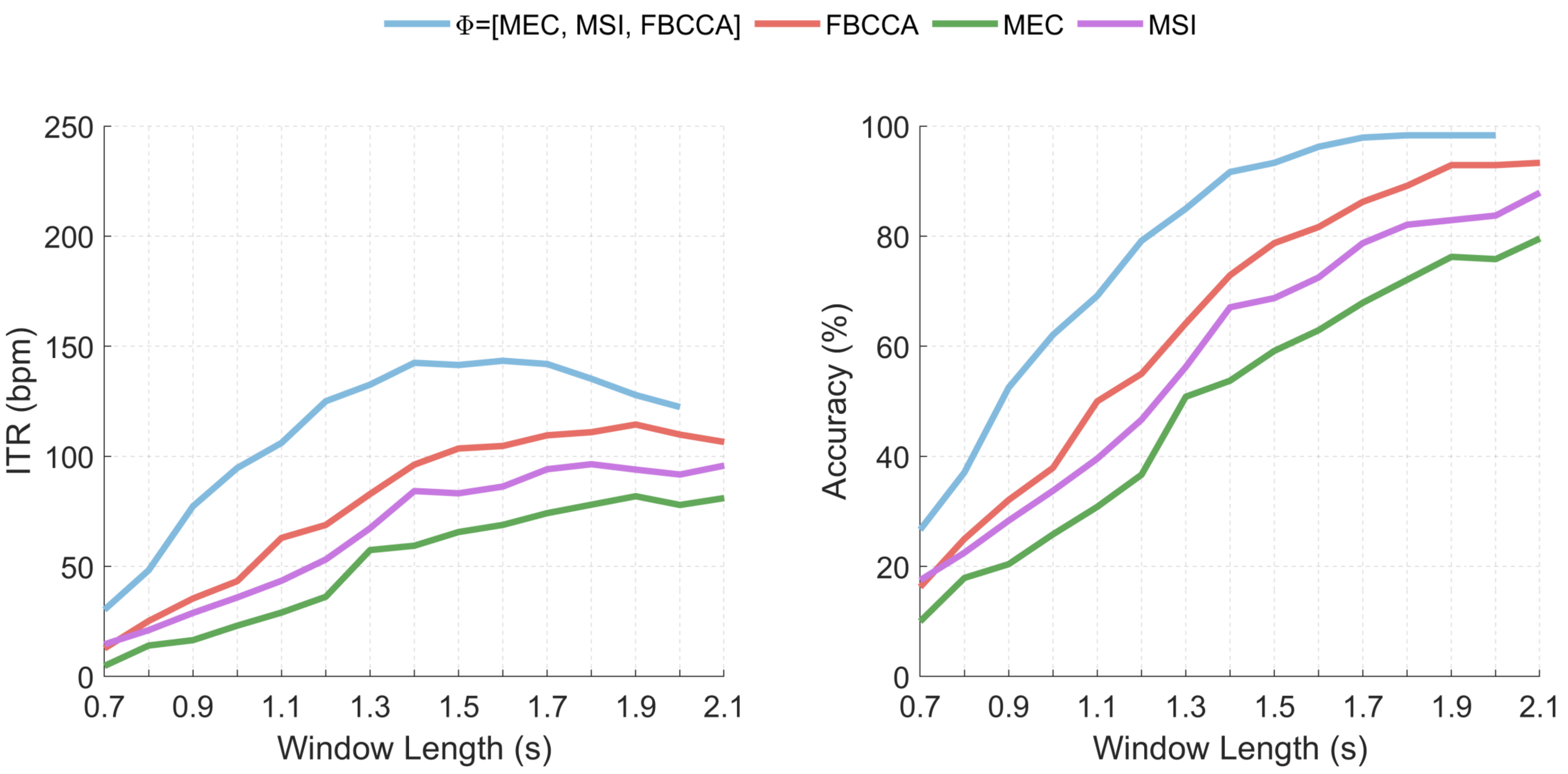

Fig. S18. (left) ITR and (right) classification accuracy for S13

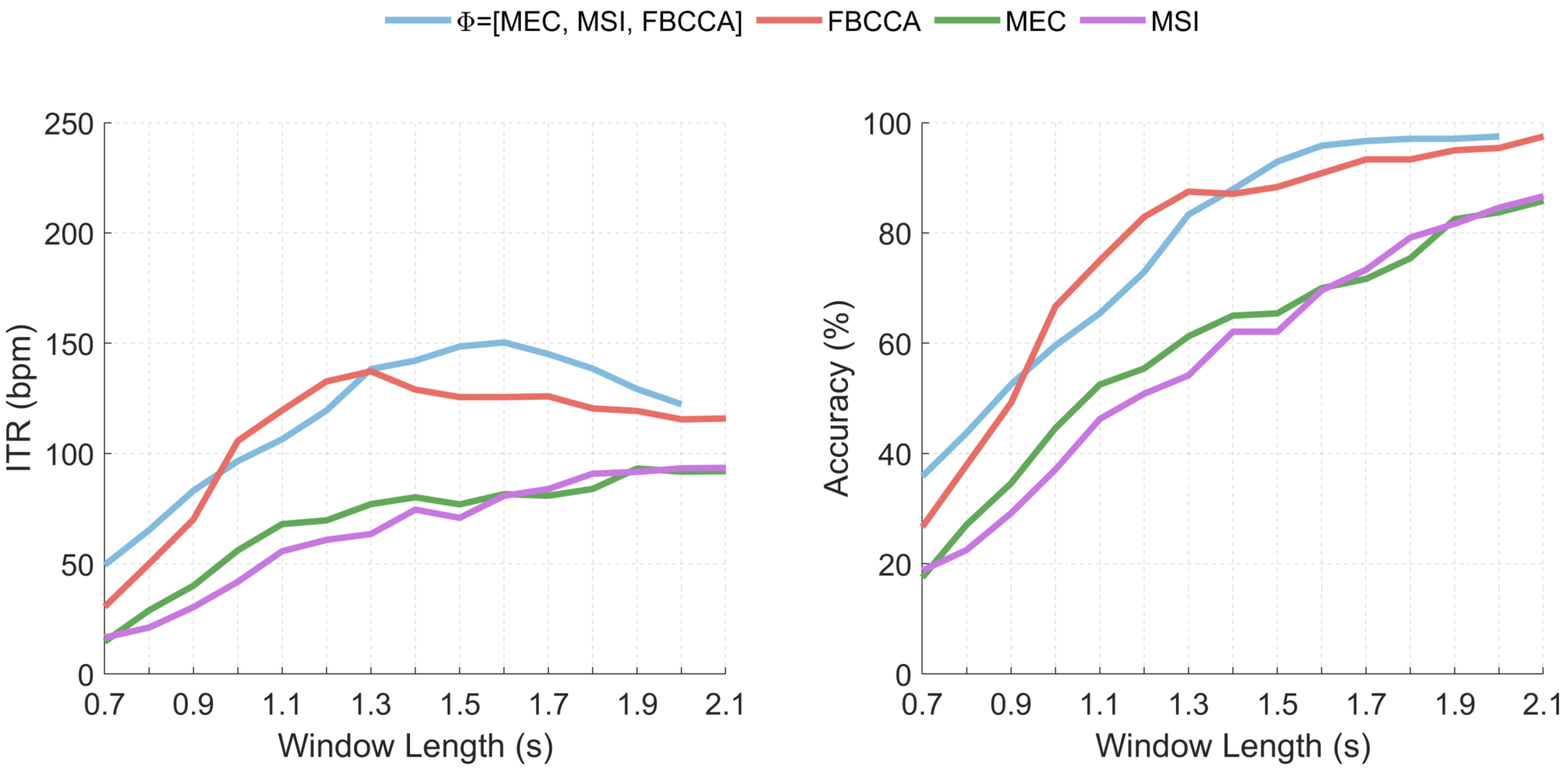

Fig. S19. (left) ITR and (right) classification accuracy for S14

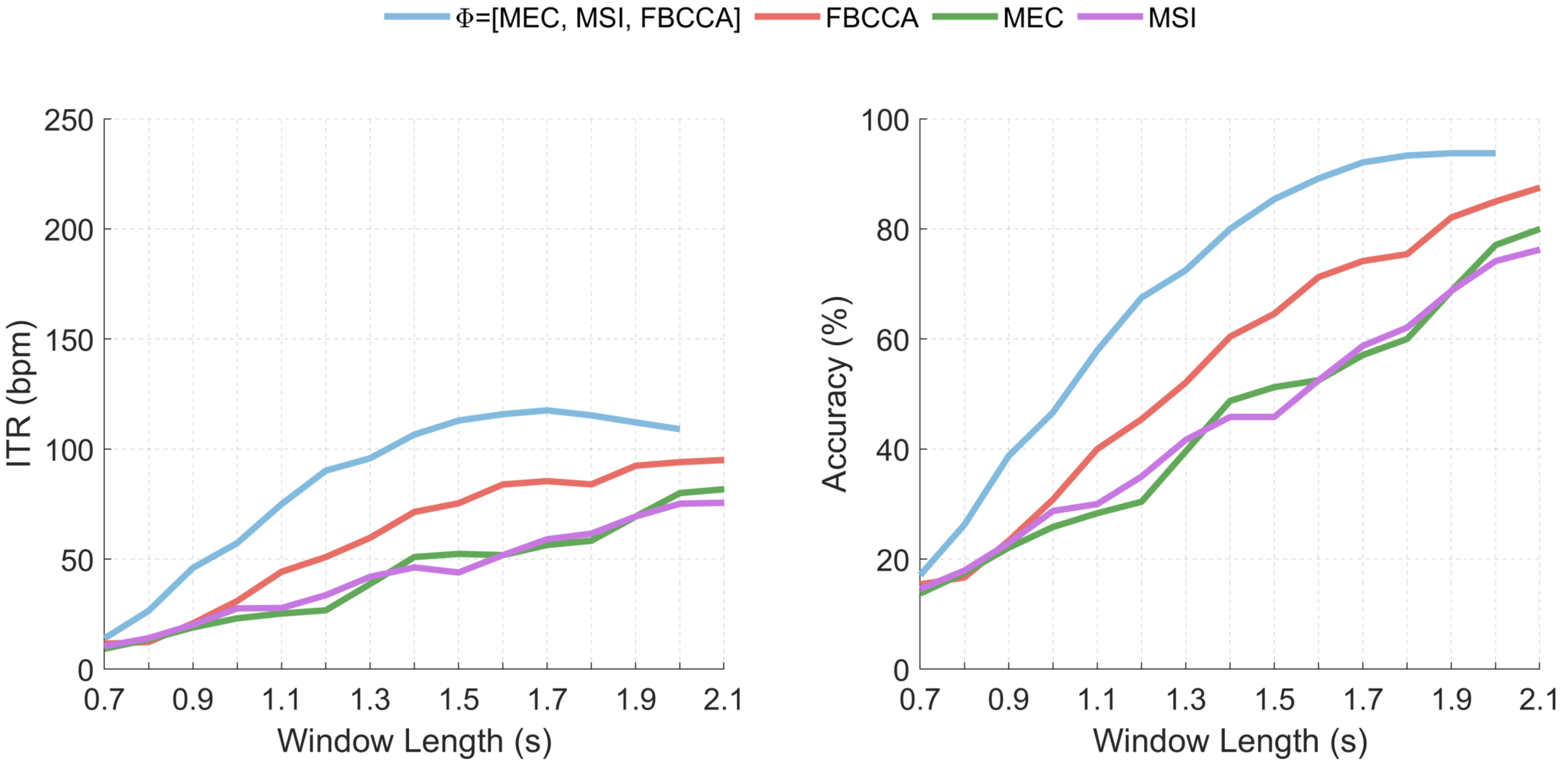

Fig. S20. (left) ITR and (right) classification accuracy for S15

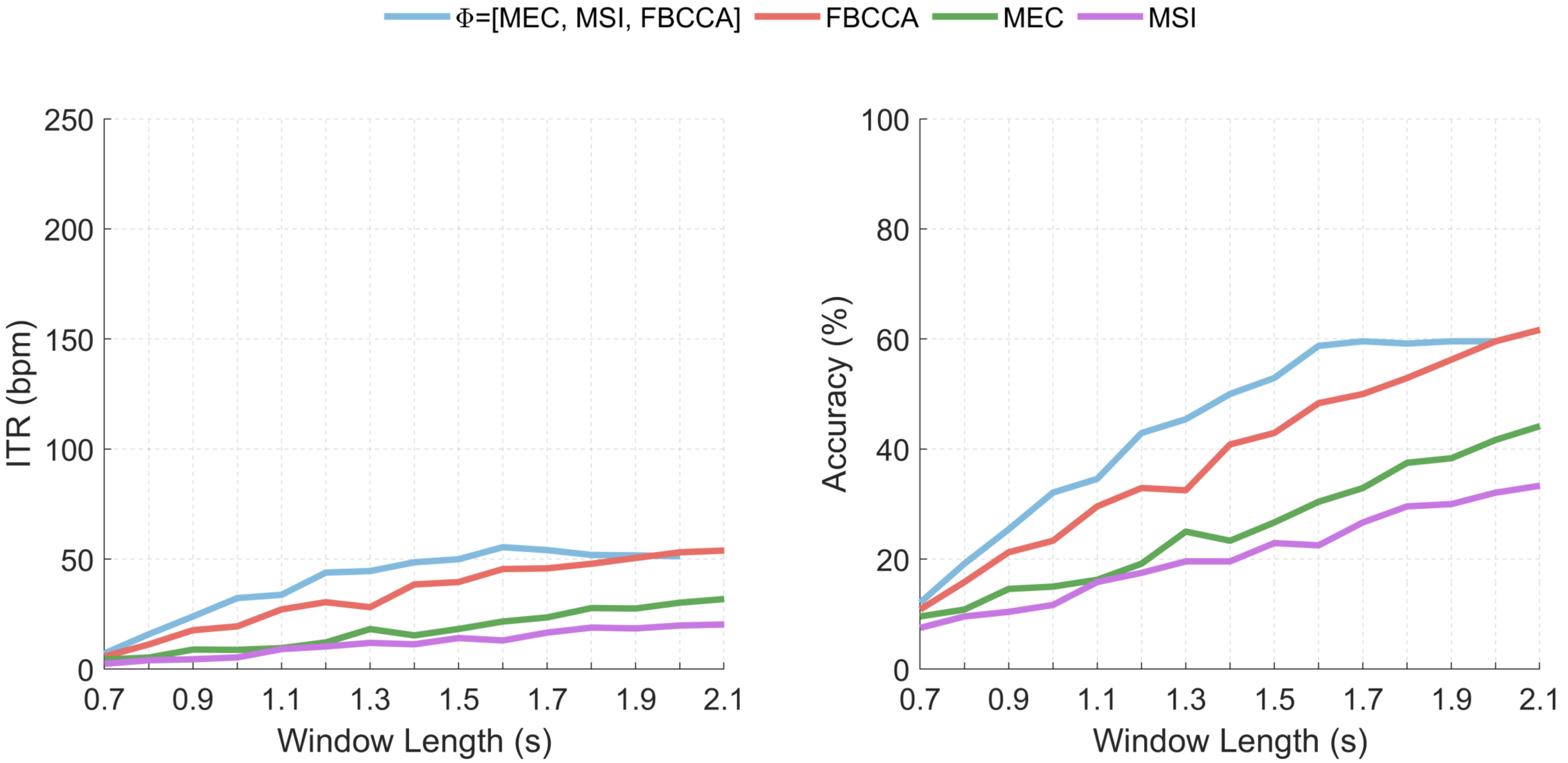

Fig. S21. (left) ITR and (right) classification accuracy for S16

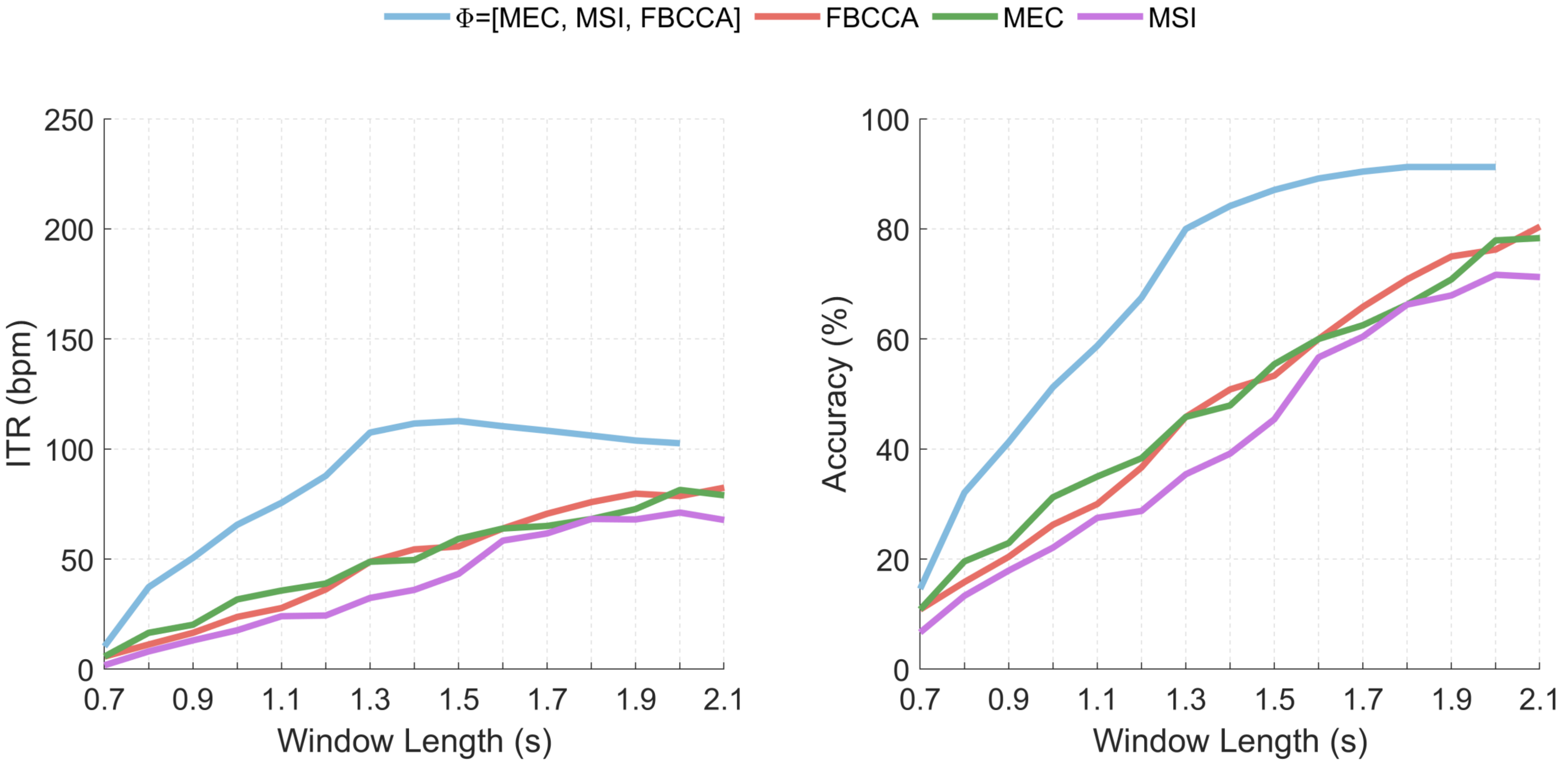

Fig. S22. (left) ITR and (right) classification accuracy for S17

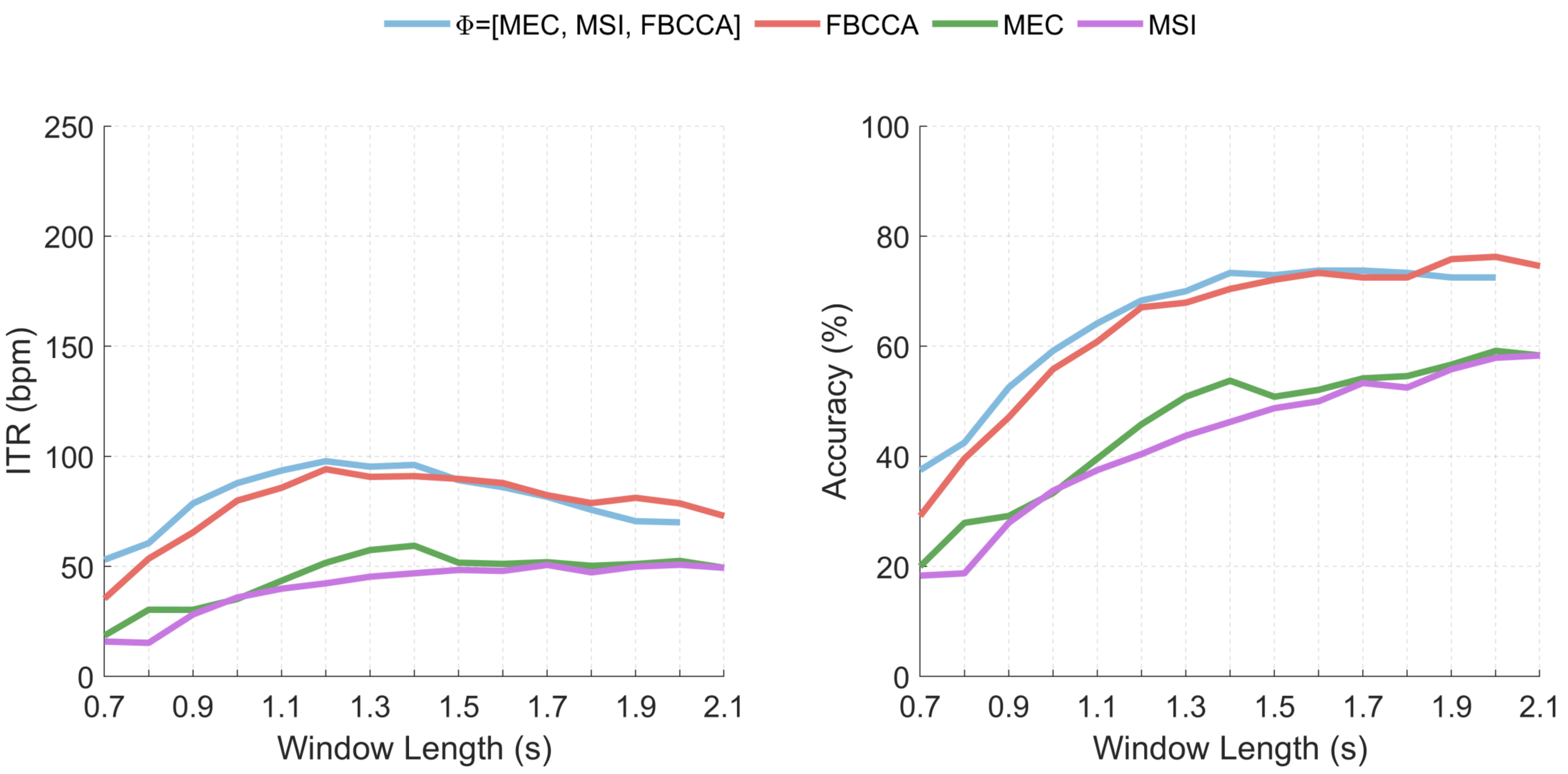

Fig. S23. (left) ITR and (right) classification accuracy for S18

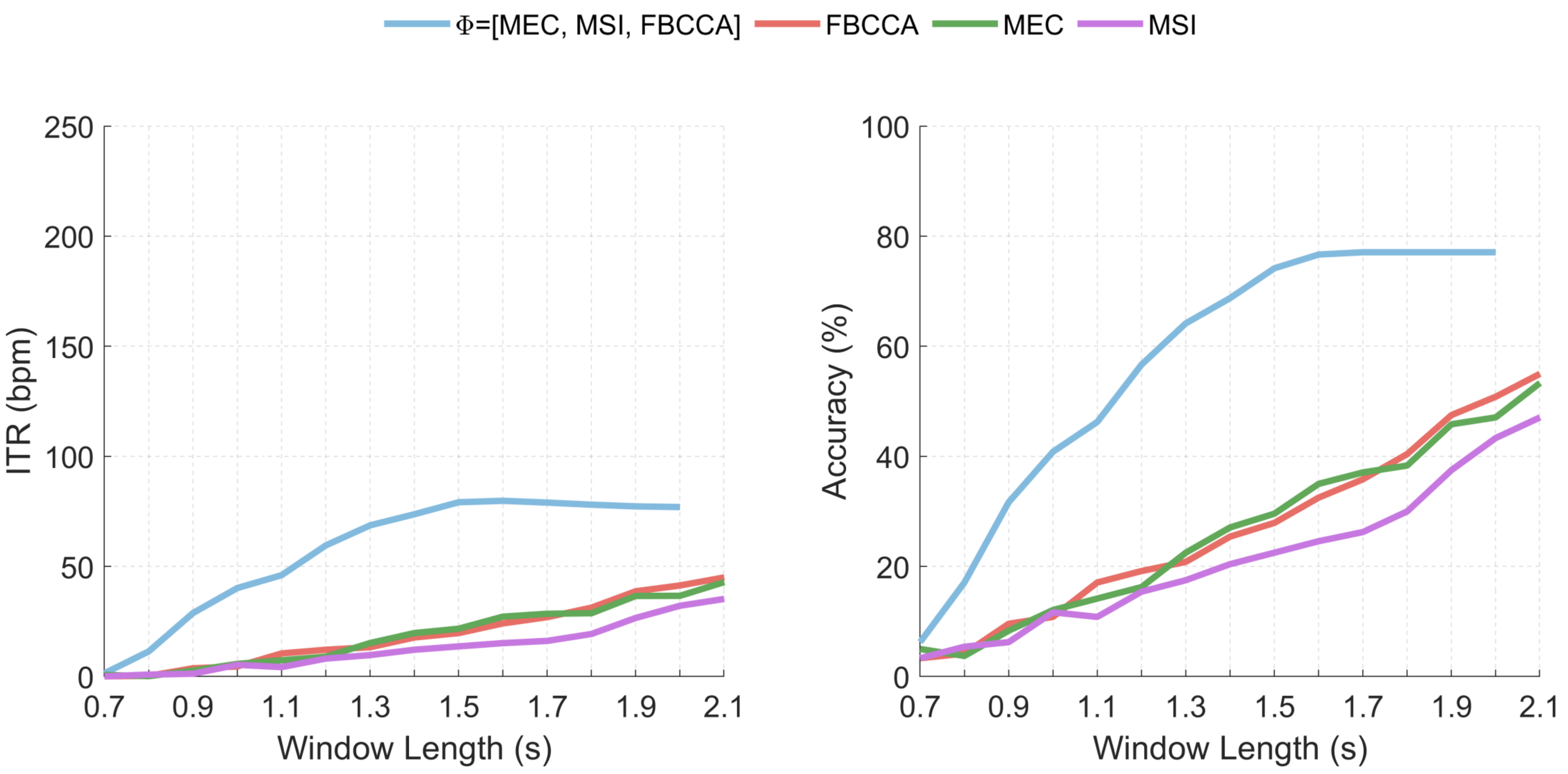

Fig. S24. (left) ITR and (right) classification accuracy for S19

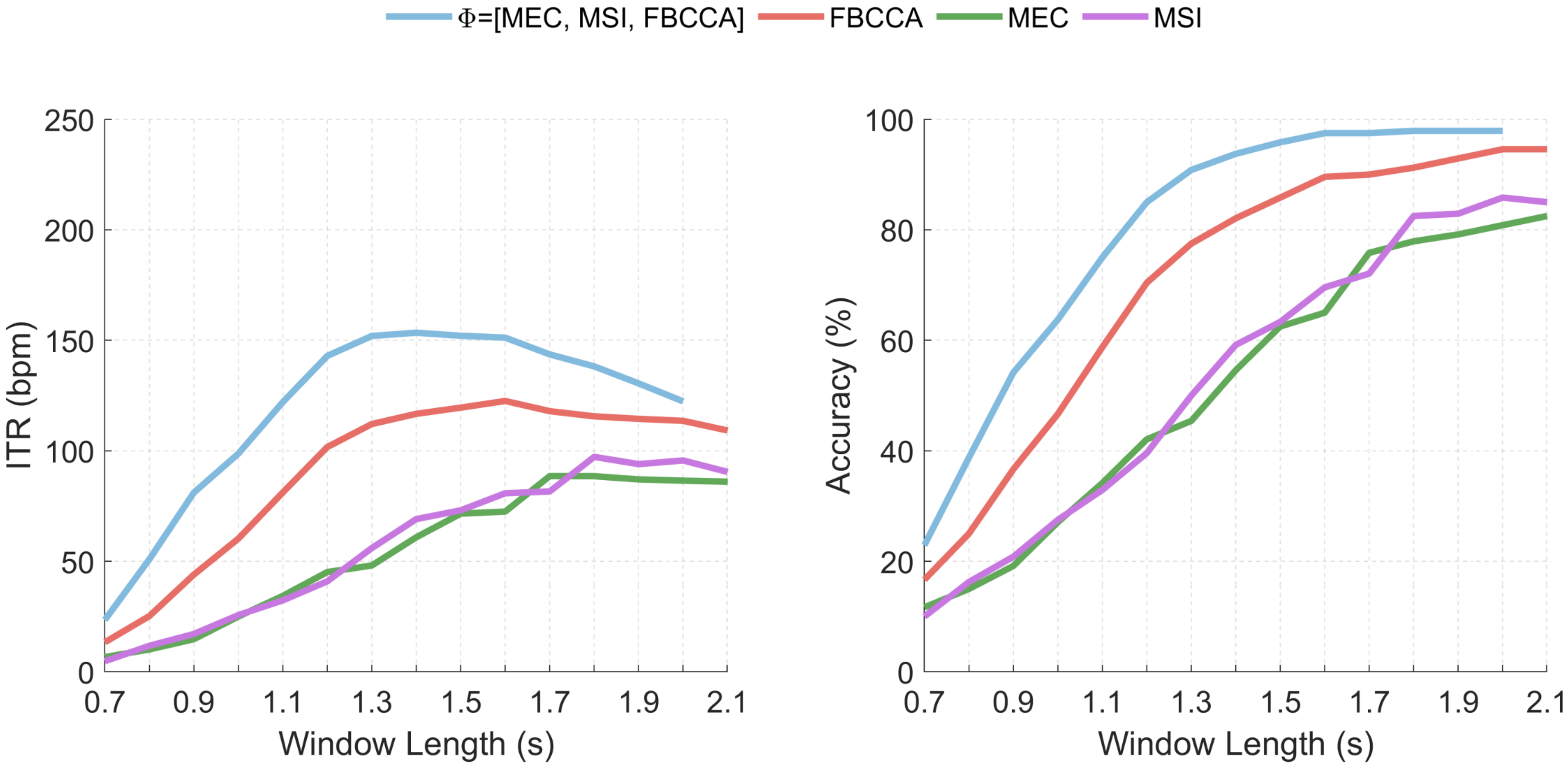

Fig. S25. (left) ITR and (right) classification accuracy for S20

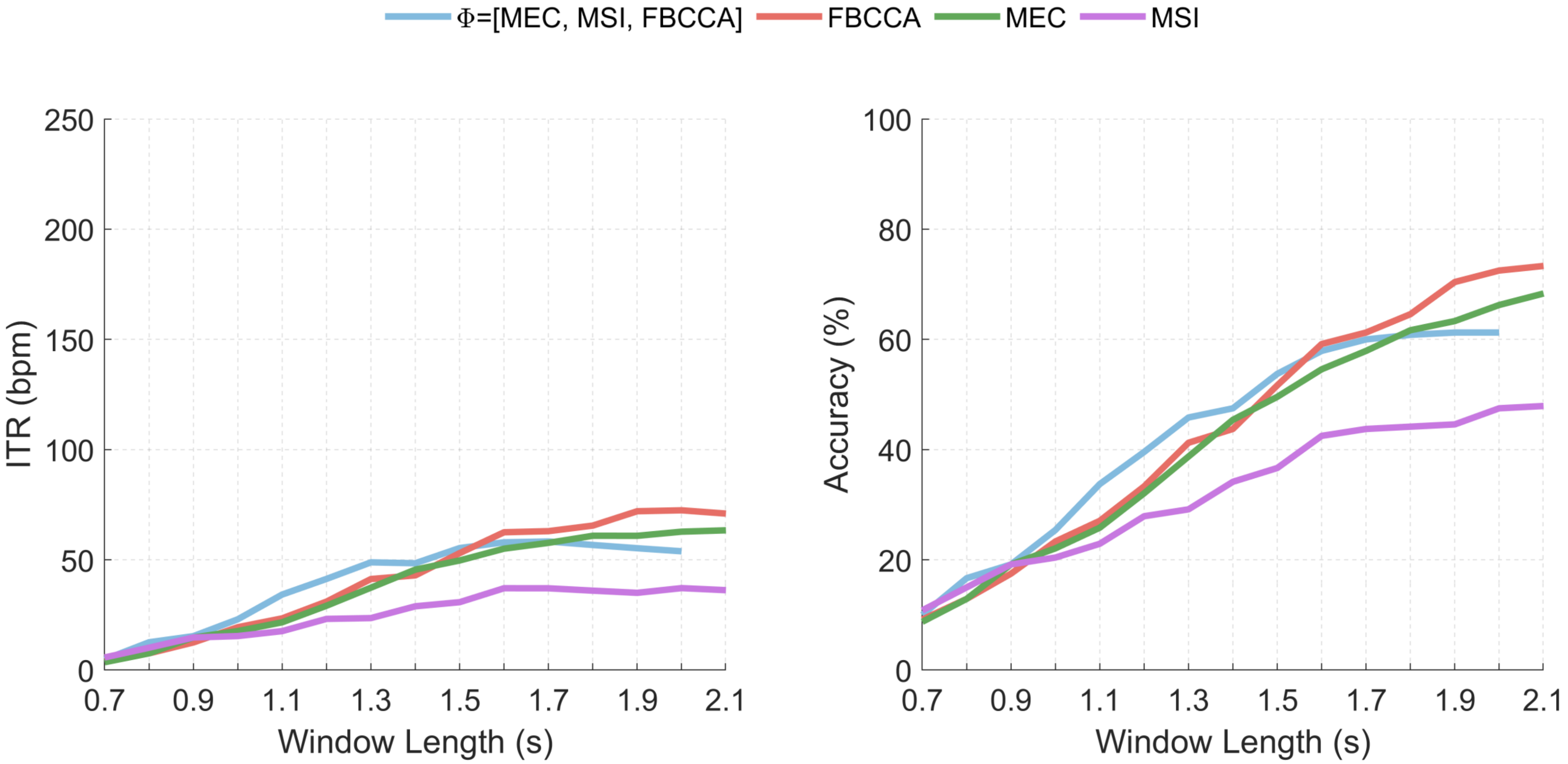

Fig. S26. (left) ITR and (right) classification accuracy for S21

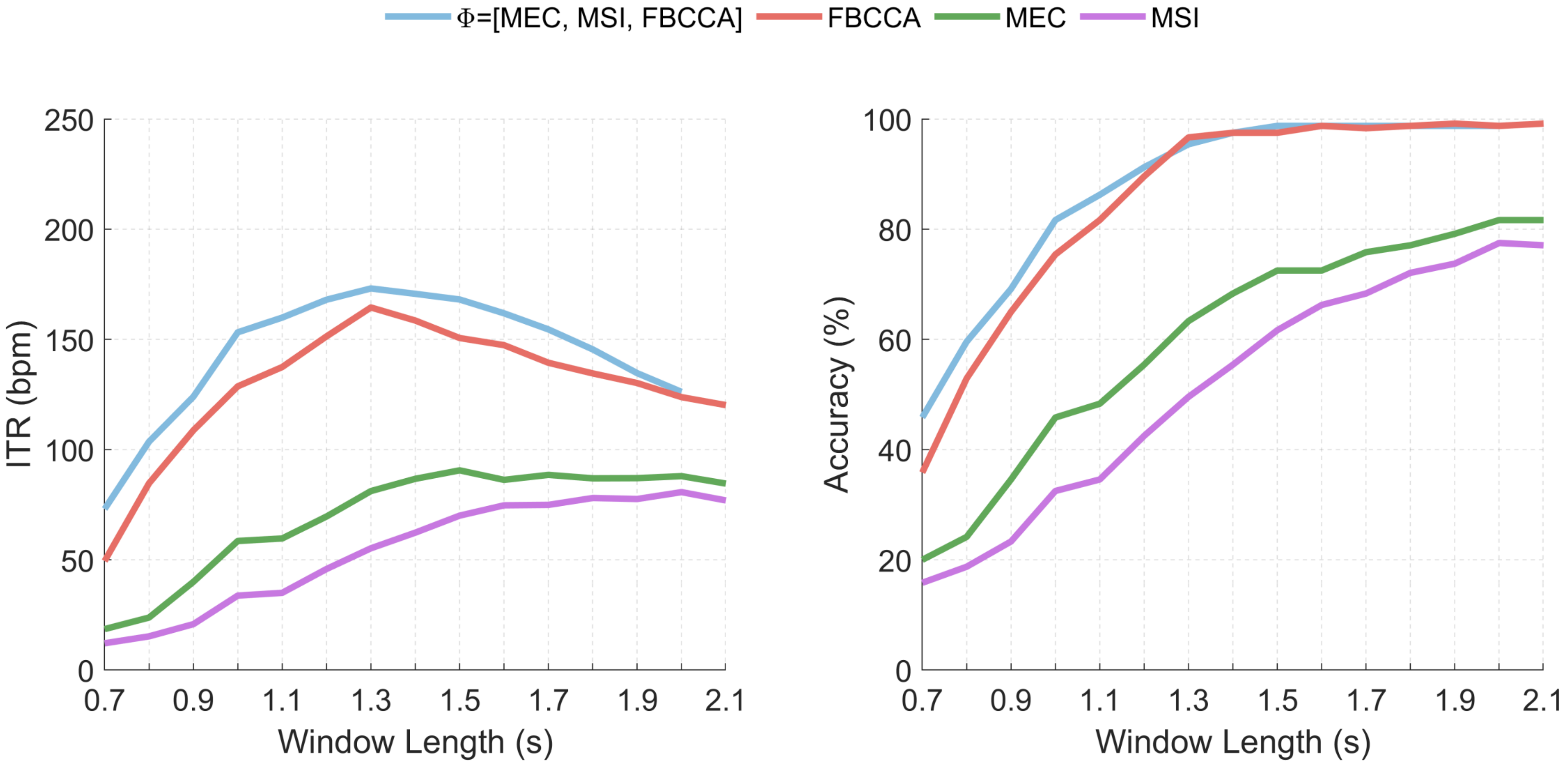

Fig. S27. (left) ITR and (right) classification accuracy for S22

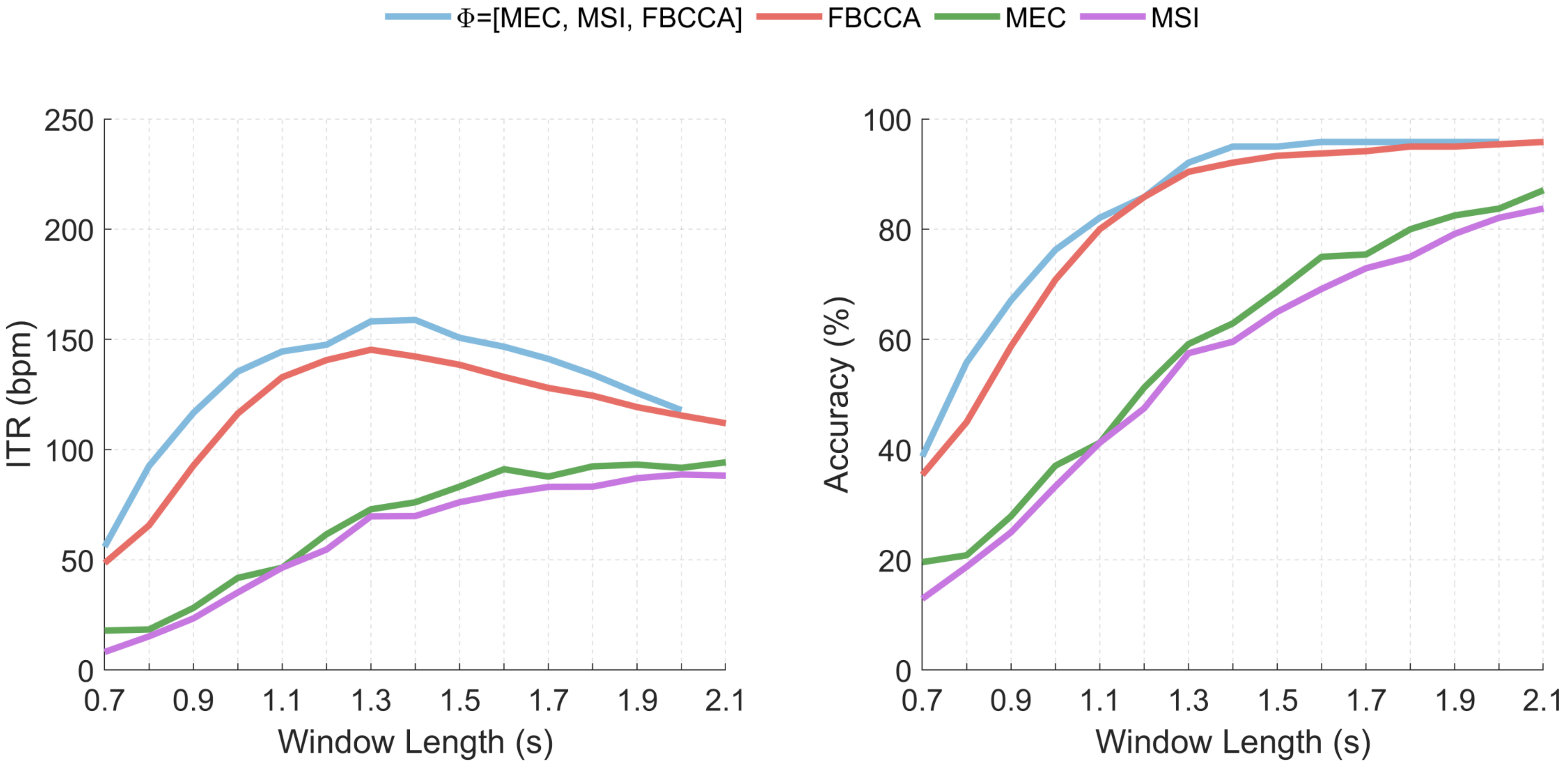

Fig. S28. (left) ITR and (right) classification accuracy for S23

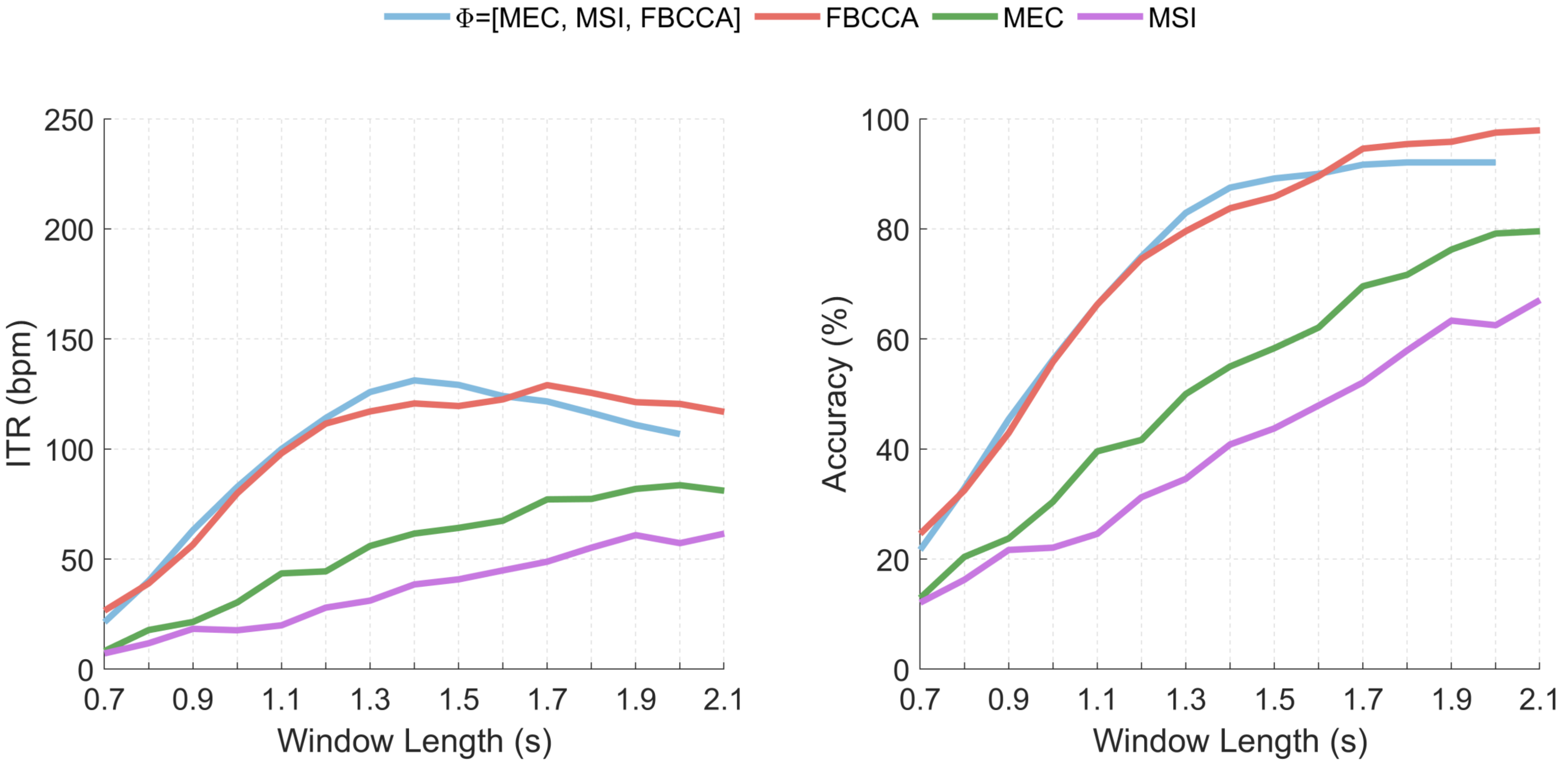

Fig. S29. (left) ITR and (right) classification accuracy for S24

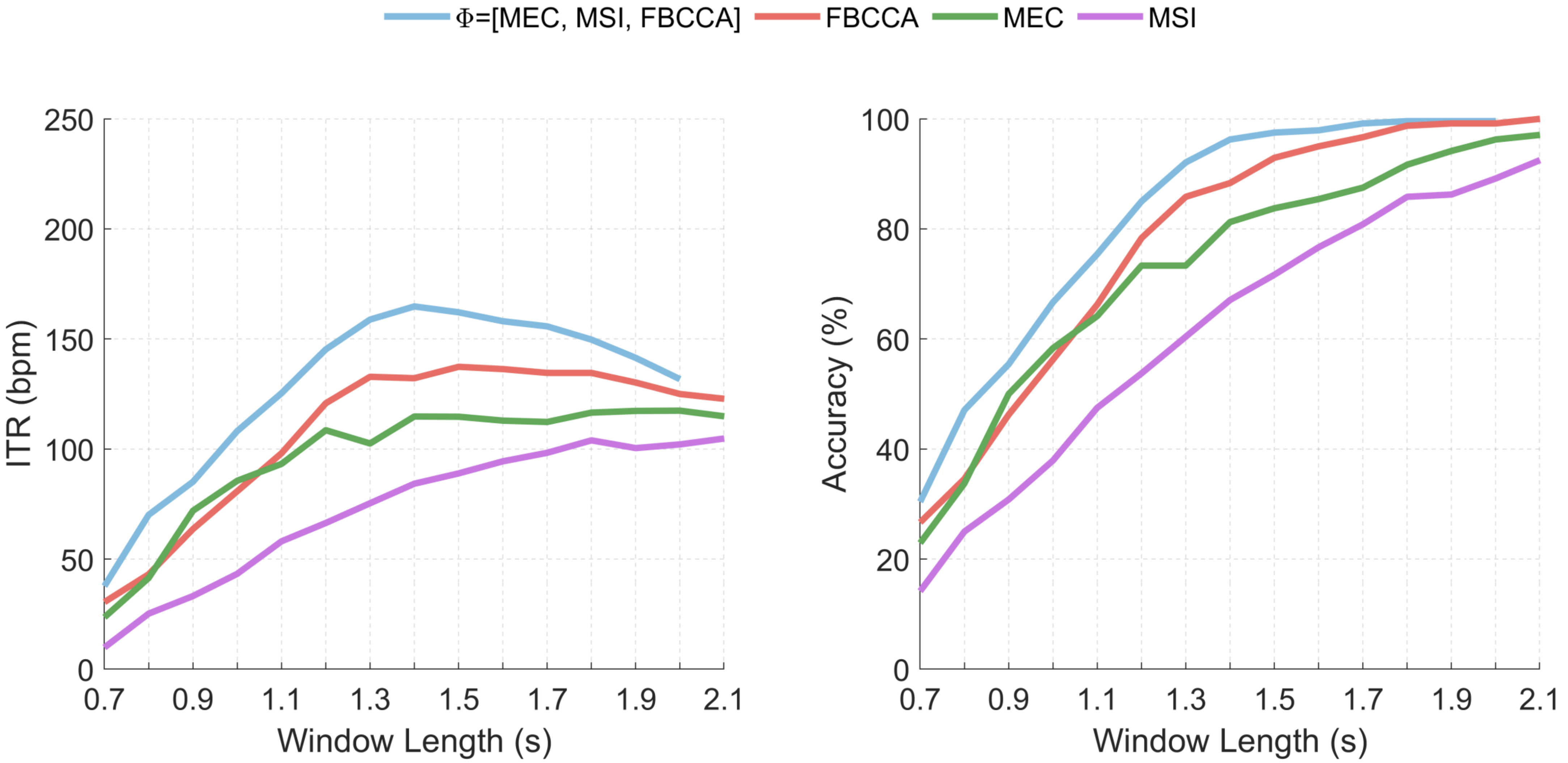

Fig. S30. (left) ITR and (right) classification accuracy for S25

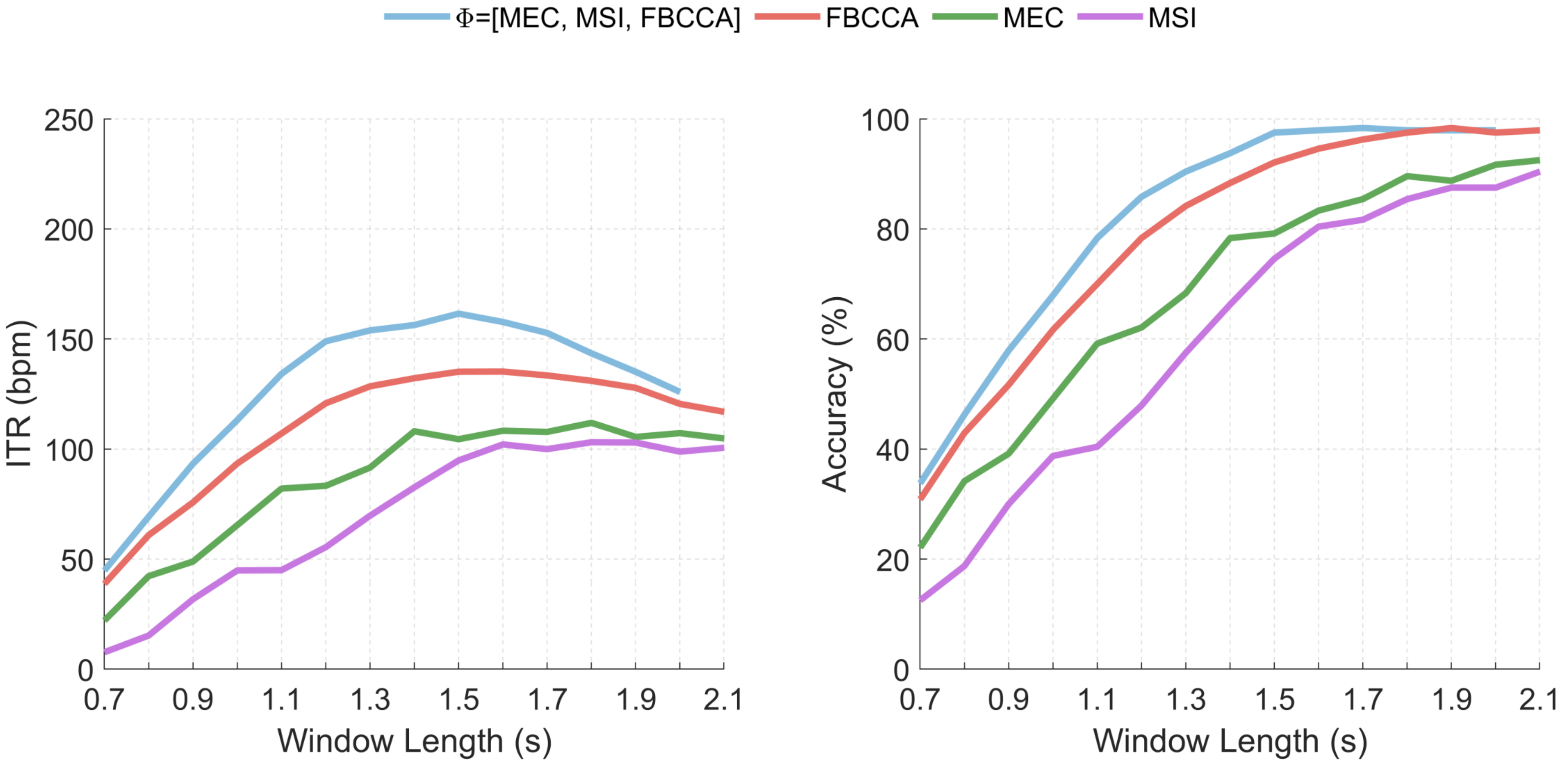

Fig. S31. (left) ITR and (right) classification accuracy for S26

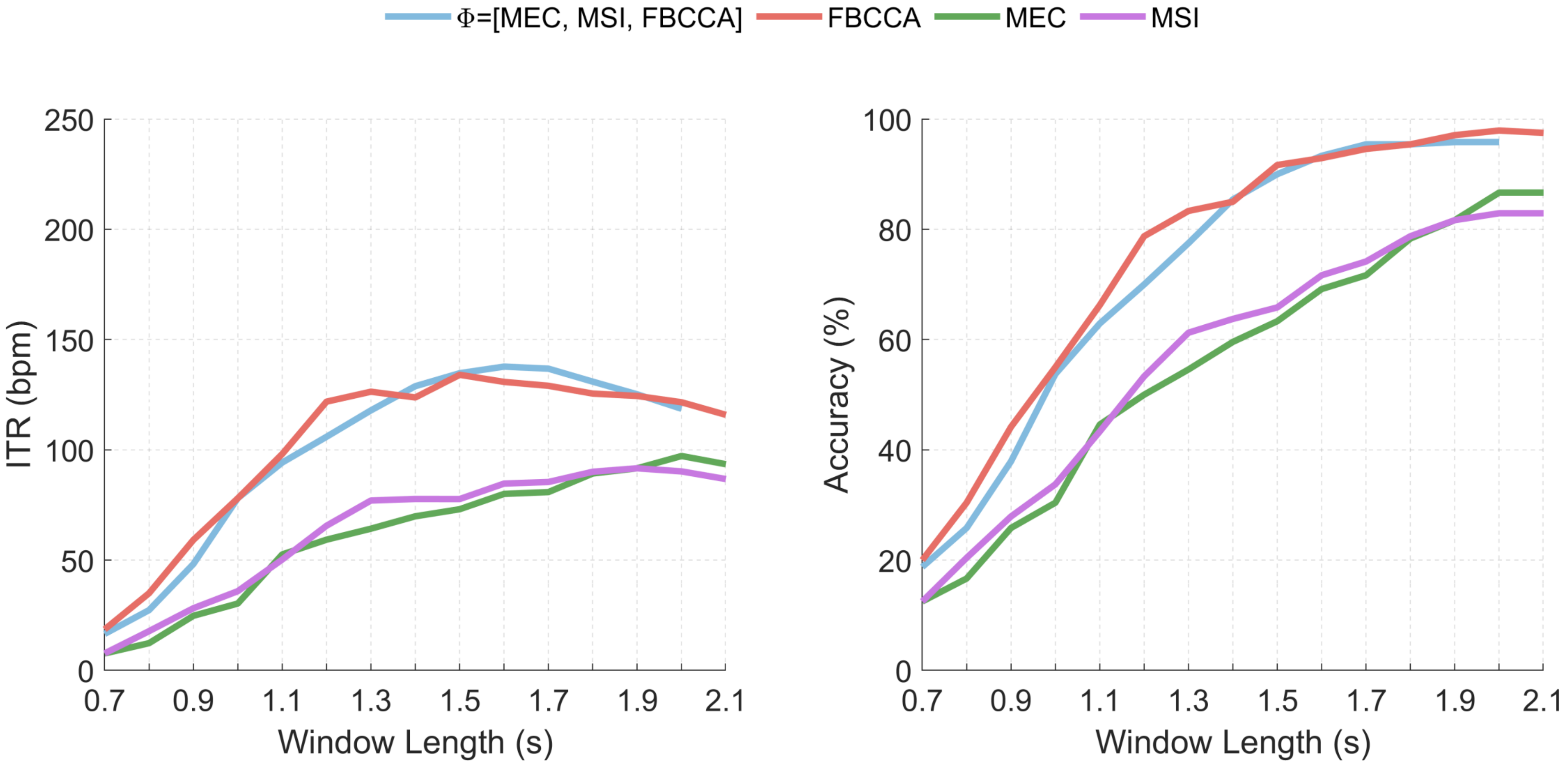

Fig. S32. (left) ITR and (right) classification accuracy for S27

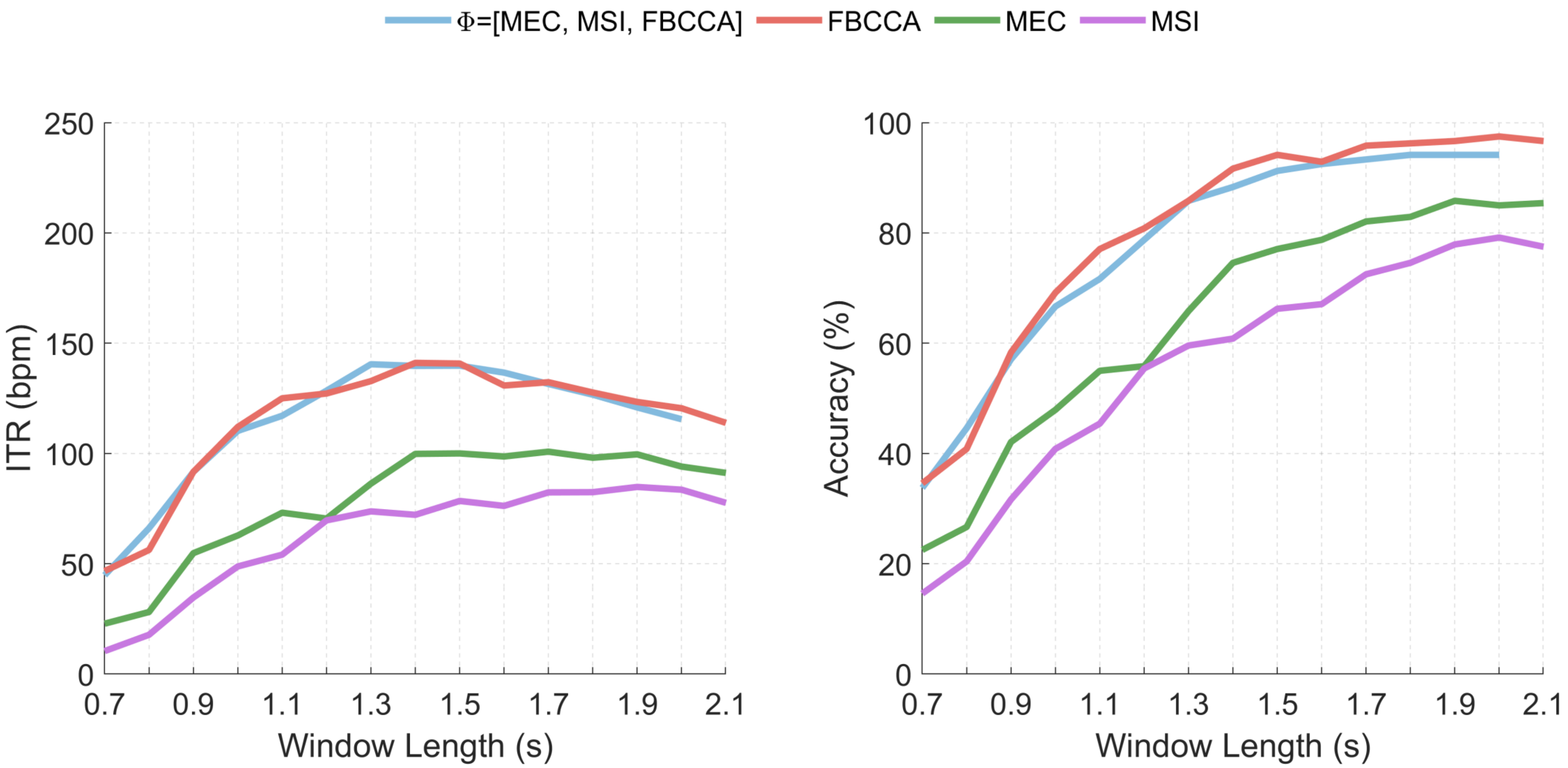

Fig. S33. (left) ITR and (right) classification accuracy for S28

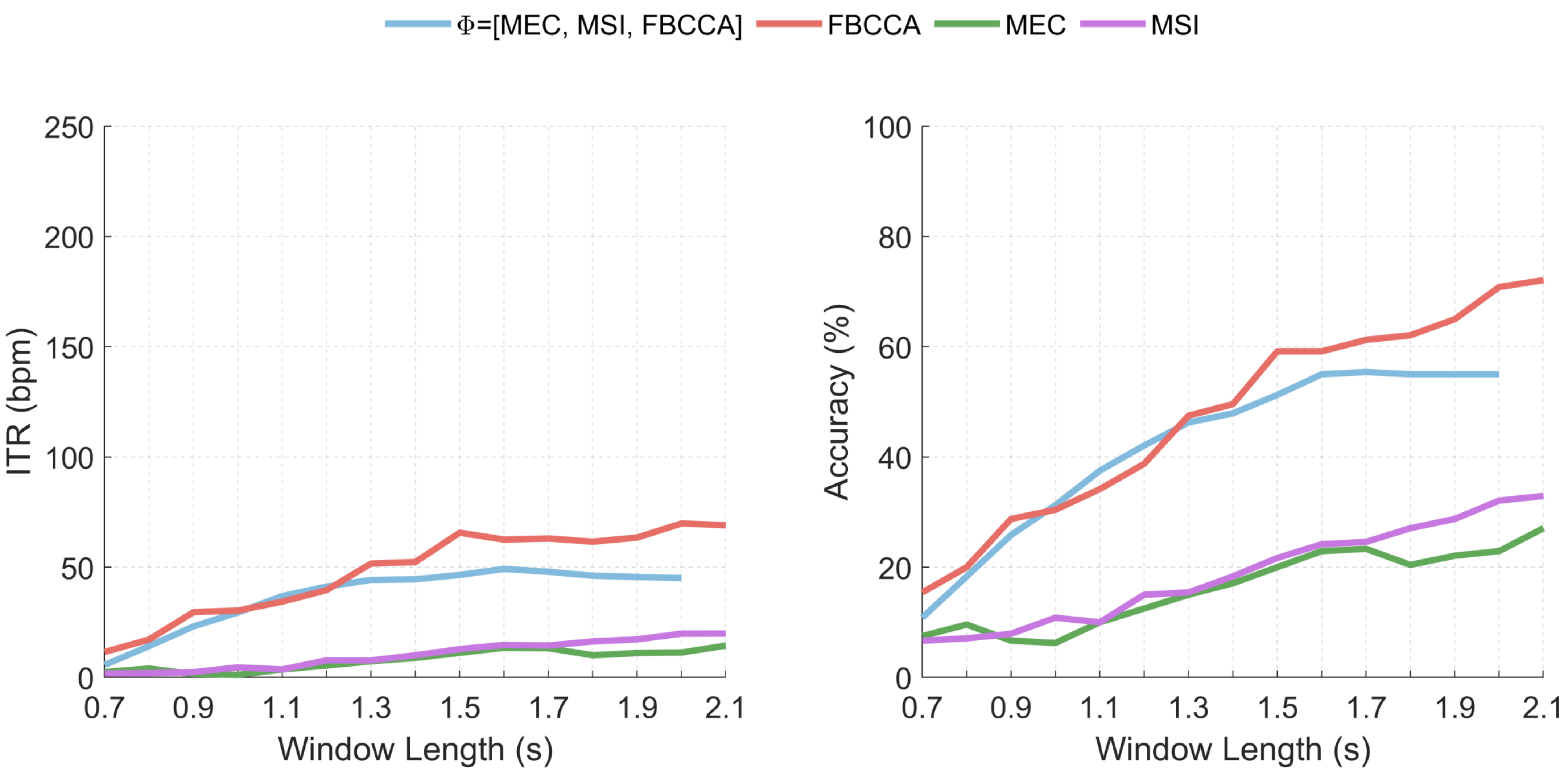

Fig. S34. (left) ITR and (right) classification accuracy for S29

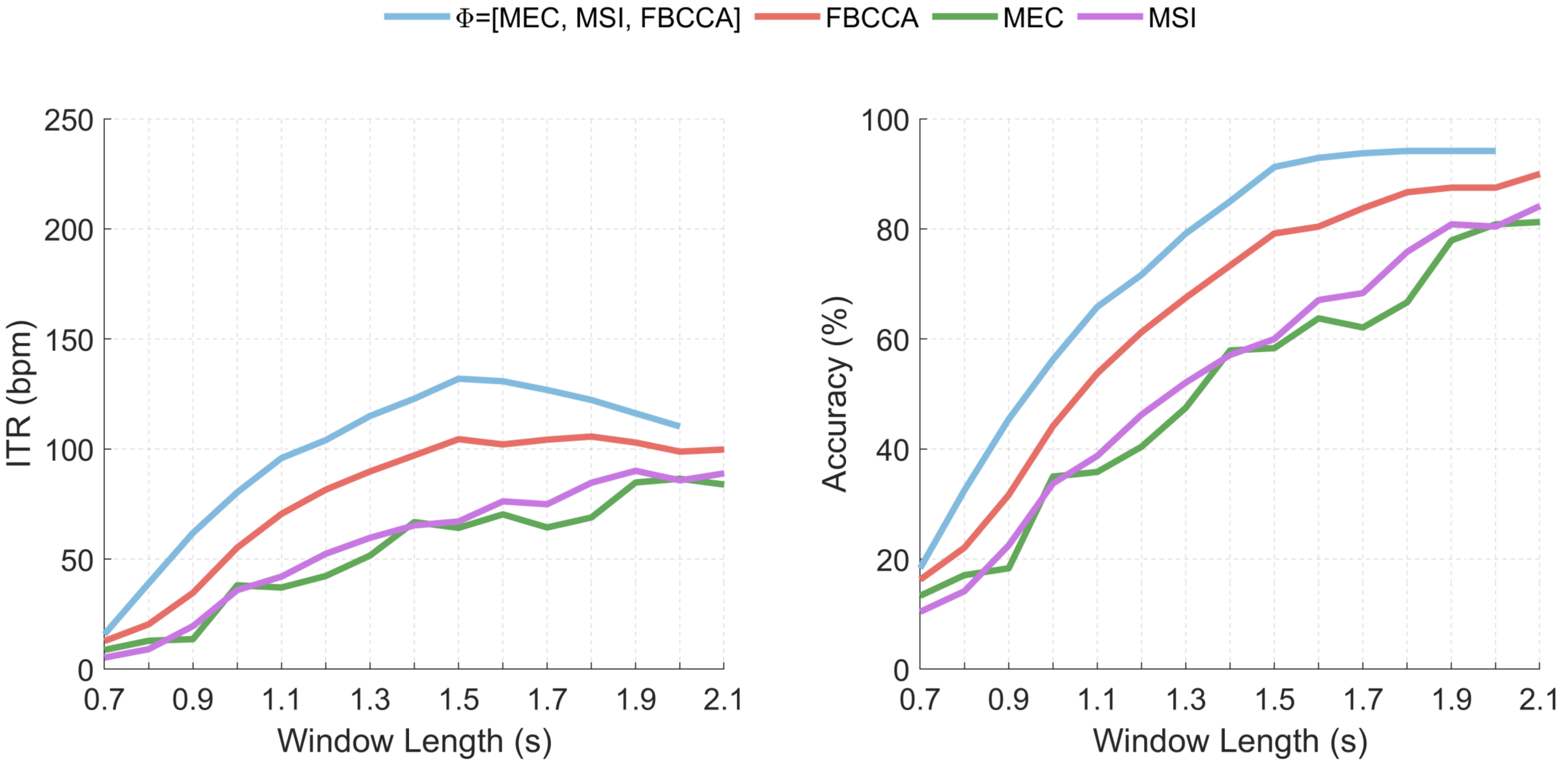

Fig. S35. (left) ITR and (right) classification accuracy for S30

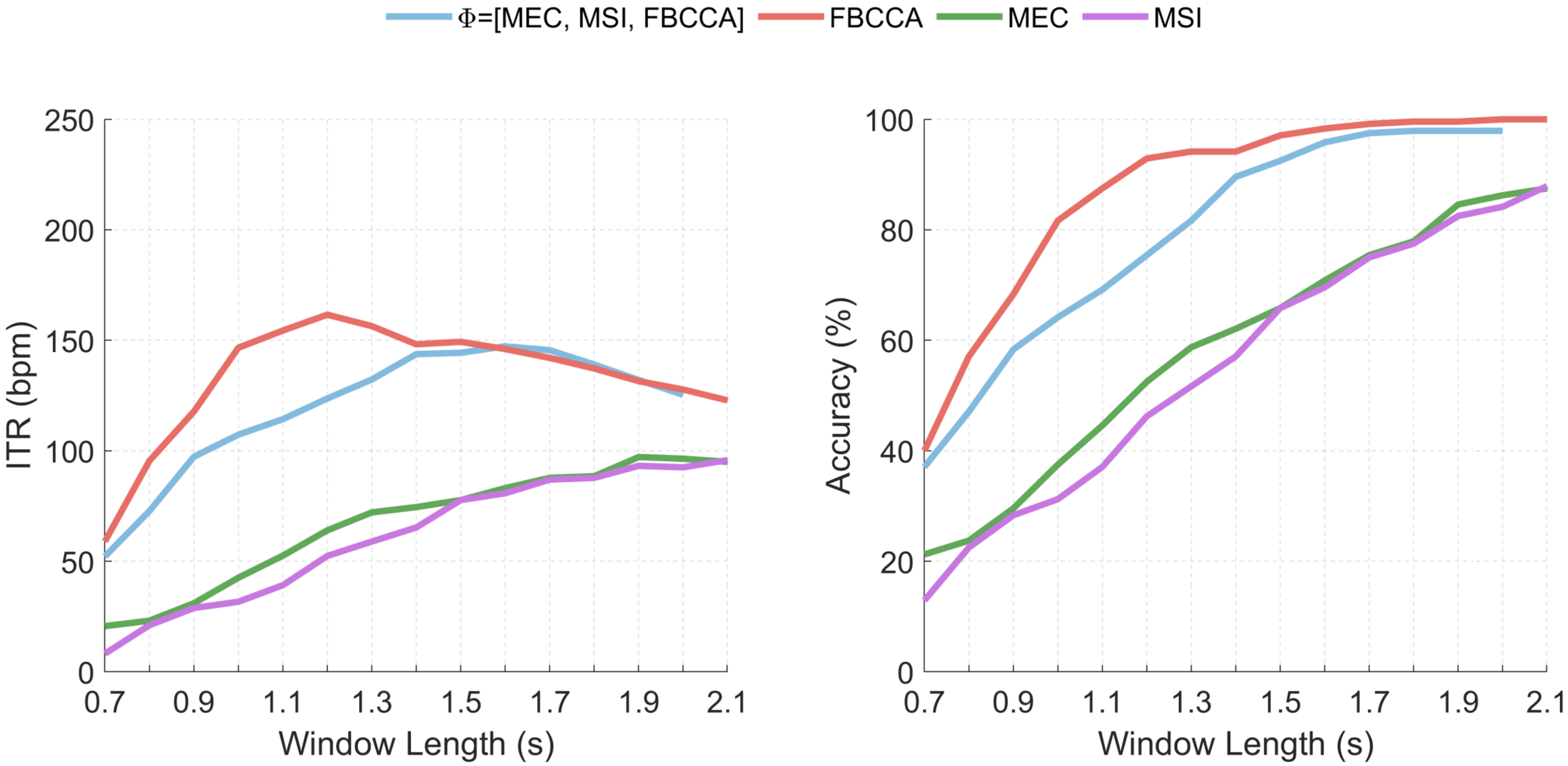

Fig. S36. (left) ITR and (right) classification accuracy for S31

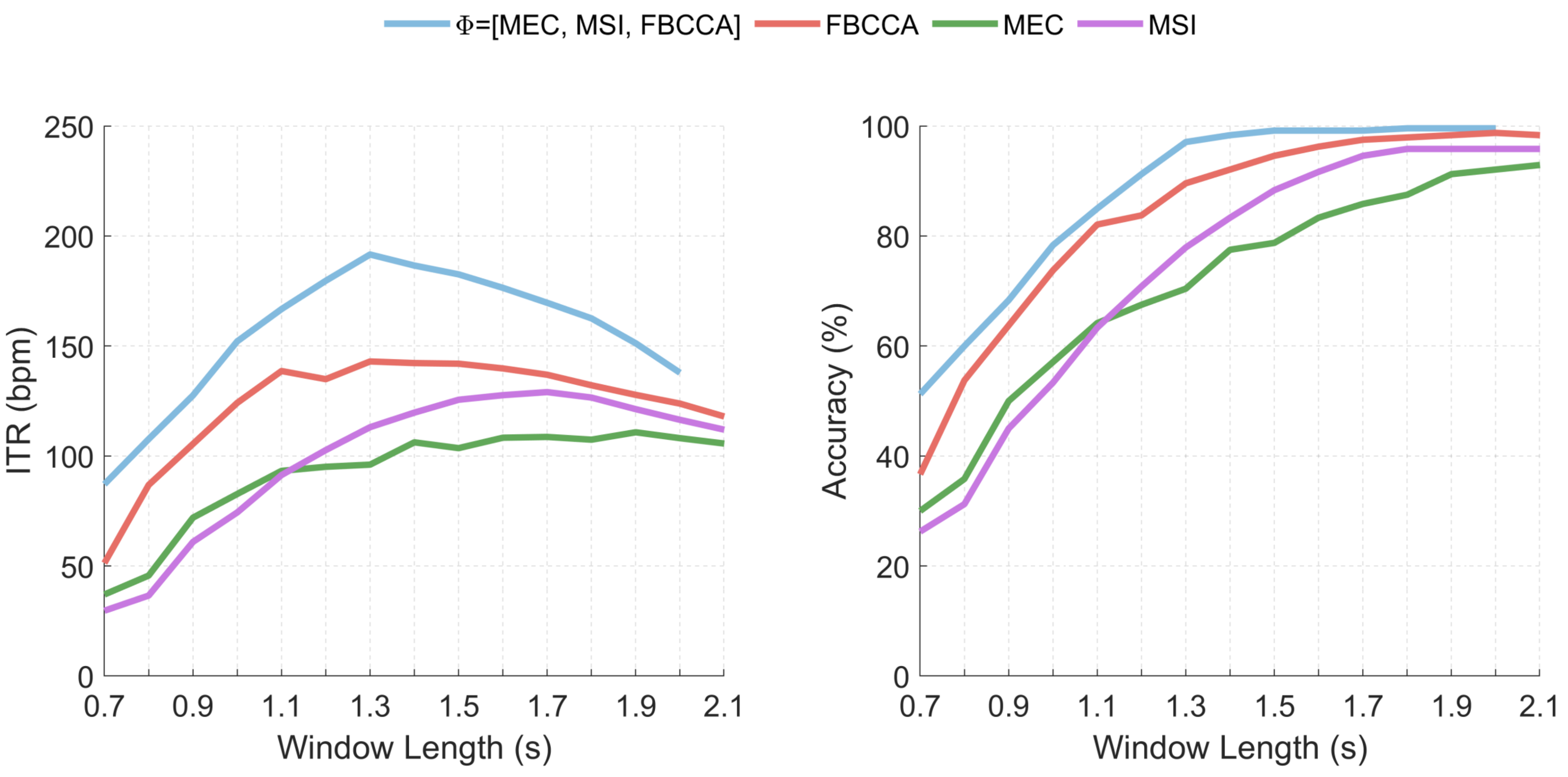

Fig. S37. (left) ITR and (right) classification accuracy for S32

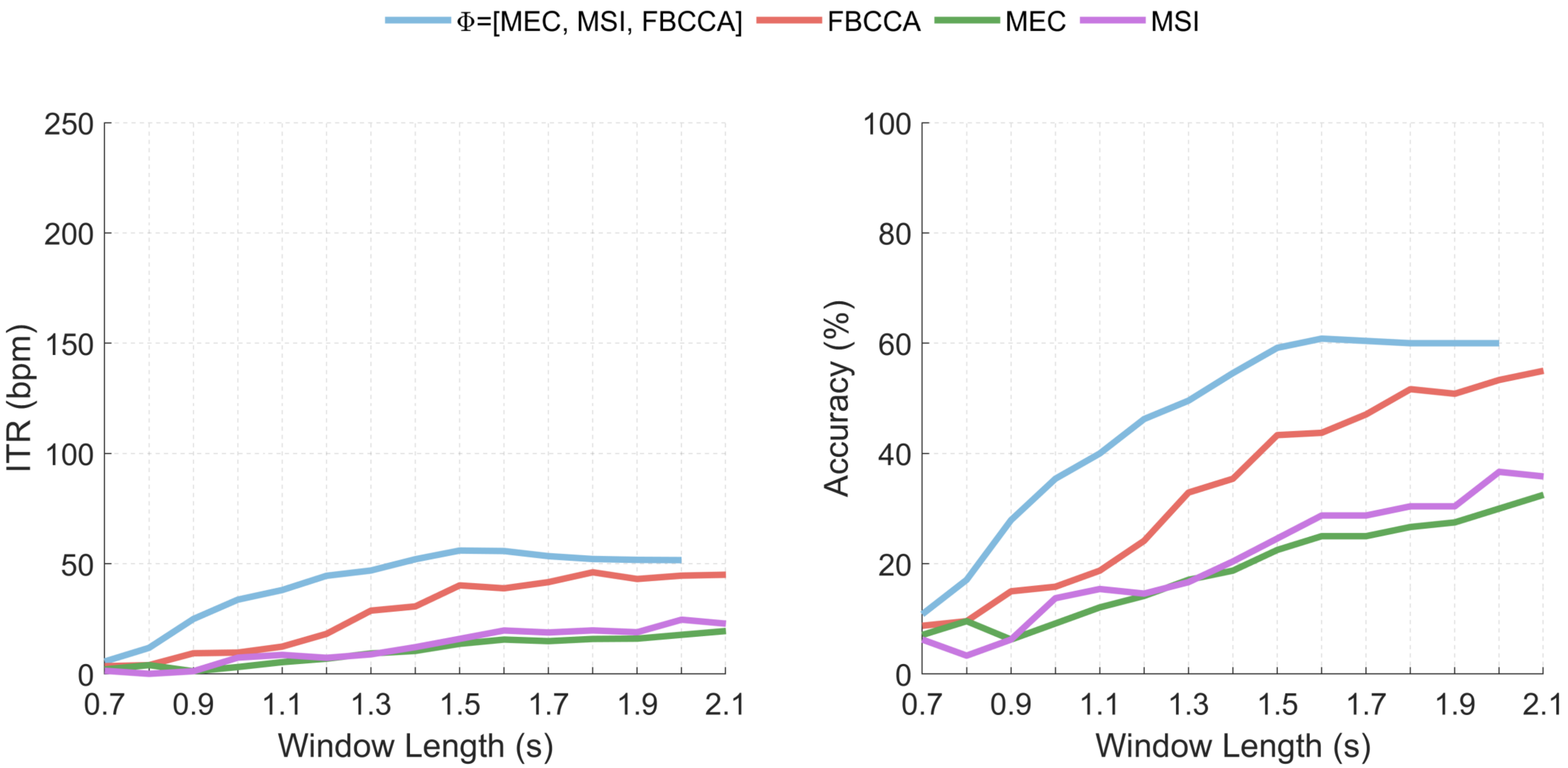

Fig. S38. (left) ITR and (right) classification accuracy for S33

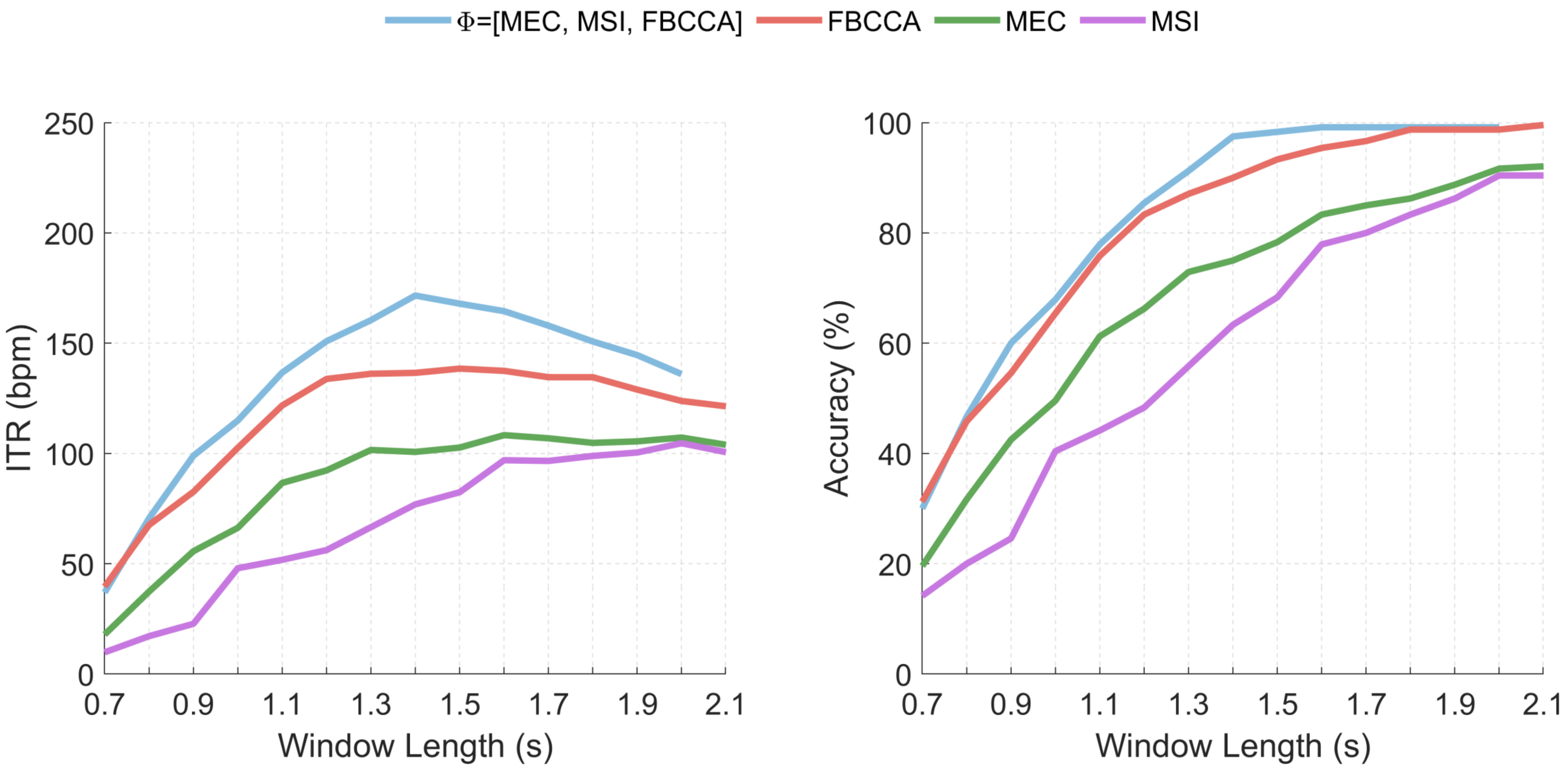

Fig. S39. (left) ITR and (right) classification accuracy for S34

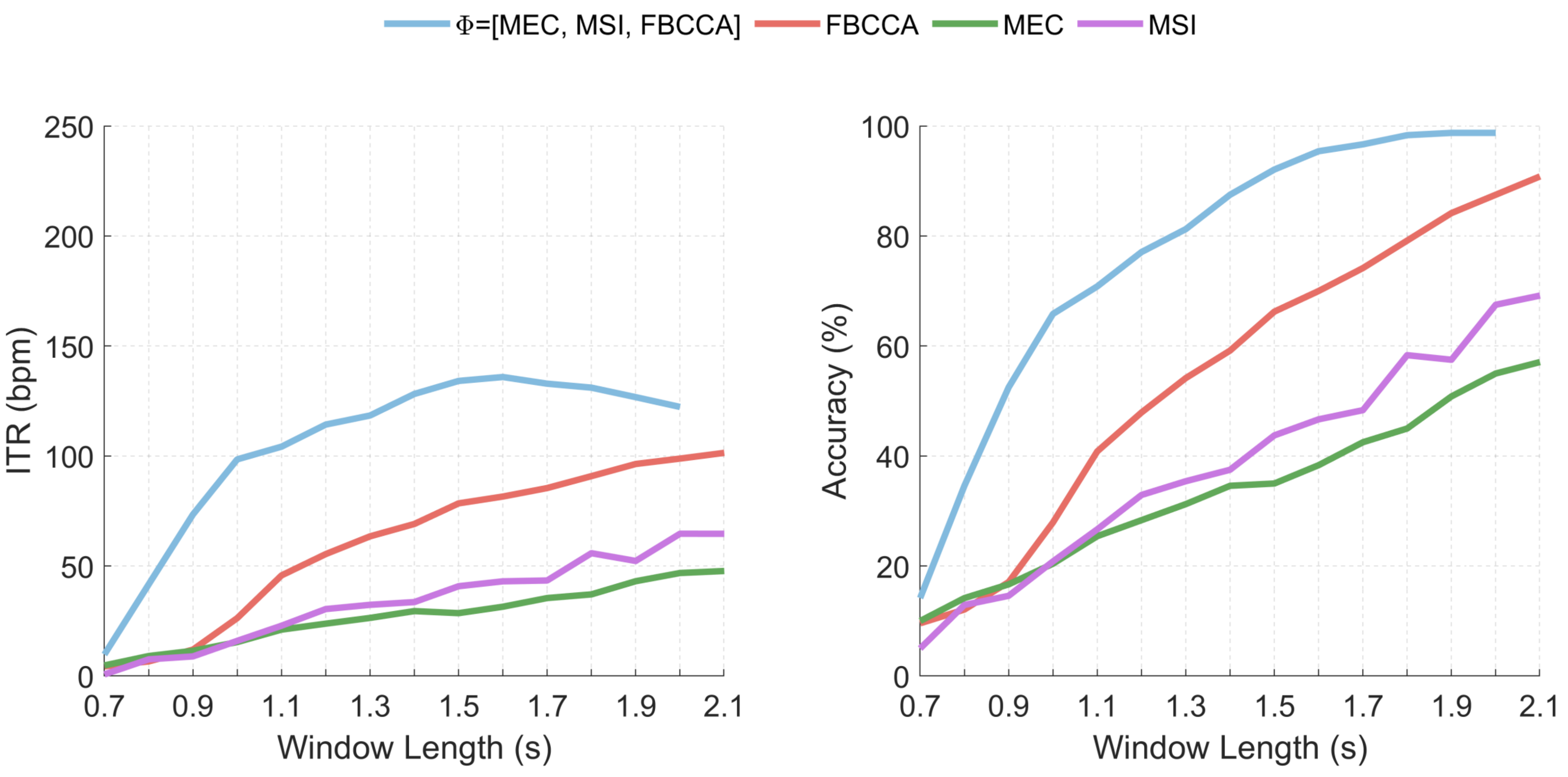

Fig. S40. (left) ITR and (right) classification accuracy for S35
